# Neoadjuvant pembrolizumab, dabrafenib and trametinib in *BRAF*^V600^-mutant resectable melanoma: the randomized phase 2 NeoTrio trial

**DOI:** 10.1038/s41591-024-03077-5

**Published:** 2024-06-21

**Authors:** Georgina V. Long, Matteo S. Carlino, George Au-Yeung, Andrew J. Spillane, Kerwin F. Shannon, David E. Gyorki, Edward Hsiao, Rony Kapoor, Jake R. Thompson, Iris Batula, Julie Howle, Sydney Ch’ng, Maria Gonzalez, Robyn P. M. Saw, Thomas E. Pennington, Serigne N. Lo, Richard A. Scolyer, Alexander M. Menzies

**Affiliations:** 1 Melanoma Institute Australia, The University of Sydney, Sydney, New South Wales Australia; 2 Faculty of Medicine and Health, The University of Sydney, Sydney, New South Wales Australia; 3 Royal North Shore Hospital, Sydney, New South Wales Australia; 4 Mater Hospital, Sydney, New South Wales Australia; 5 Charles Perkins Centre, The University of Sydney, Sydney, New South Wales Australia; 6 Westmead Hospital, Westmead, New South Wales Australia; 7 Blacktown Hospital, Blacktown, New South Wales Australia; 8 Peter MacCallum Cancer Centre, Melbourne, Victoria Australia; 9 Sir Peter MacCallum Department of Oncology, The University of Melbourne, Melbourne, Victoria Australia; 10 Royal Prince Alfred Hospital, Sydney, New South Wales Australia; 11 Chris O’Brien Lifehouse, Sydney, New South Wales Australia; 12 Concord Repatriation Hospital, Concord, New South Wales Australia; 13 I-MED Radiology Network, Mater Hospital, Sydney, New South Wales Australia; 14 NSW Health Pathology, Sydney, New South Wales Australia

**Keywords:** Melanoma, Health sciences

## Abstract

Immune checkpoint inhibitors and BRAF-targeted therapy each improve survival in melanoma. Immune changes early during targeted therapy suggest the mechanisms of each drug class could work synergistically. In the non-comparative, randomized, phase 2 NeoTrio trial, we investigated whether targeted therapy could boost the proportion of patients achieving long-term recurrence-free survival with neoadjuvant immunotherapy in resectable stage III *BRAF*^V600^-mutant melanoma. Sixty patients (42% females) were randomized to pembrolizumab alone (*n* = 20), sequential therapy (dabrafenib plus trametinib followed by pembrolizumab; *n* = 20) or concurrent (triple) therapy (*n* = 20), followed by surgery and adjuvant therapy. The primary outcome was pathological response; secondary outcomes included radiographic response, recurrence-free survival, overall survival, surgical outcomes, peripheral blood and tumor analyses and safety. The pathological response rate was 55% (11/20; including six pathological complete responses (pCRs)) with pembrolizumab, 50% (10/20; three pCRs) with sequential therapy and 80% (16/20; ten pCRs) with concurrent therapy, which met the primary outcome in each arm. Treatment-related adverse events affected 75–100% of patients during neoadjuvant treatment, with seven early discontinuations (all in the concurrent arm). At 2 years, event-free survival was 60% with pembrolizumab, 80% with sequential therapy and 71% with concurrent therapy. Recurrences after major pathological response were more common in the targeted therapy arms, suggesting a reduction in response ‘quality’ when targeted therapy is added to neoadjuvant immunotherapy. Risking the curative potential of immunotherapy in melanoma cannot be justified. Pending longer follow-up, we suggest that immunotherapy and targeted therapy should not be combined in the neoadjuvant setting for melanoma. ClinicalTrials.gov registration: NCT02858921.

## Abstract (web summary)

In this non-comparative trial, patients with *BRAF*^V600^-mutant resectable melanoma received either pembrolizumab alone, a sequential combination of pembrolizumab, dabrafenib and trametinib, or a concurrent combination thereof, showing encouraging clinical response rates in the concurrent therapy arm and awaiting longer follow-up.

## Main

*BRAF* mutations are found in ~40% of cutaneous melanomas^1^ and are associated with more aggressive early-stage disease and a younger patient population^2,3^. BRAF and mitogen-activated protein kinase kinase (MEK)-targeted therapies and immune checkpoint inhibitor (ICI) immunotherapy have led to remarkable improvements in response and survival outcomes in the advanced (unresectable stage III or IV melanoma) setting^4,5^. Almost 70% of patients achieve an objective response with targeted therapy; however, most progress within 2 years^5^. While ICIs have a lower response rate, acquired resistance is much less common^4^.

Treatment with BRAF/MEK inhibitors has been shown to induce a favorable tumor immune microenvironment in melanoma^6–8^. In a study of metastatic melanomas, there was a substantial increase in tumor-infiltrating CD4^+^ and CD8^+^ T cells after 7 days of treatment with a BRAF inhibitor (vemurafenib or dabrafenib)^6^. The infiltrate persisted with the addition of a MEK inhibitor (trametinib) to dabrafenib, which was also associated with an increase in CD4^+^, CD8^+^ and PD-1^+^ T cells, as well as in programmed death-ligand 1 (PD-L1) expression on tumor cells^8^. In another study, treatment with 10–14 days of BRAF inhibitor alone (vemurafenib) or with concurrent MEK inhibition (dabrafenib plus trametinib) resulted in a marked increase in CD8^+^ T cells and an increase in PD-L1 expression^7^. In a melanoma model, MEK1/MEK2 inhibition was associated with the reprogramming of CD8^+^ T cells into a potent stemness state^9^. Anti-programmed cell death-protein 1 (PD-1) ICI treatment is most effective in patients where T cells can recognize the tumor, and the highest response rates are found in patients with a higher density of tumor-infiltrating lymphocytes^10–13^, suggesting that BRAF/MEK inhibitor treatment could amplify the antitumor activity of anti-PD-1. However, the most effective way to combine these treatments is unknown.

Different approaches to combining BRAF-targeted therapy and ICIs have been examined in the setting of advanced melanoma. Three trials (Keynote-022, COMBI-i and IMspire150) have tested the use of BRAF-targeted therapy with or without concurrent anti-PD-1 in advanced *BRAF*-mutant melanoma^14–16^. In all three studies, addition of anti-PD-1 prolonged progression-free survival but substantially increased toxicity^14–16^. Two additional trials (SECOMBIT and DREAMseq) have investigated sequencing options, identifying a combination of anti-PD-1 and anti-CTLA-4 to progression followed by BRAF-targeted therapy as the optimal regimen in a clinical trial-eligible population^17,18^.

For patients with resectable melanoma, presurgical or ‘neoadjuvant’ drug therapy is an area of active investigation. In a pooled analysis of neoadjuvant trials, a pathological complete response (pCR) was achieved by 47% of patients with neoadjuvant BRAF-targeted therapy^19,20^ and 33% with neoadjuvant ICI^21–24^ (20% with anti-PD-1 monotherapy and 43% with combination anti-PD-1 plus anti-CTLA-4)^25^. Interestingly, the 2-year recurrence-free survival (RFS) rate was only 79% in patients achieving a pCR to targeted therapy, compared with 96% for patients achieving any pathological response (pCR, near-pathological complete response (near-pCR) or pathological partial response (pPR)) to an ICI regimen^25^, suggesting (alongside 5-year outcomes from the NeoCombi trial^26^) that neoadjuvant targeted therapy has no benefit over adjuvant targeted therapy. This is in contrast to ICI, where in the phase II SWOG S1801 trial, the 2-year event-free survival (EFS) rate was 72% with neoadjuvant (plus adjuvant) anti-PD-1 versus 49% with adjuvant anti-PD-1 alone (the current standard of care)^27^. It is possible that combining neoadjuvant anti-PD-1 with targeted therapy could enhance the impressive, sometimes curative, gains made with neoadjuvant ICI even further.

The NeoTrio trial (ClinicalTrials.gov registration: NCT02858921) was designed to determine whether targeted therapy (dabrafenib plus trametinib) enhances the benefit of neoadjuvant anti-PD-1 (pembrolizumab) in stage III resectable *BRAF*-mutant melanoma. Specifically, we investigated pathological response and survival outcomes with sequential or concurrent BRAF-targeted therapy and anti-PD-1, or anti-PD-1 alone, in the 6 weeks before surgery. Here we present the primary endpoint of pathological response rate and key secondary endpoints, namely radiographic response, RFS, overall survival (OS), surgical outcomes and safety. Additional planned secondary endpoints not reported in this paper are peripheral blood and tumor analyses.

## Results

### Baseline patient characteristics and disposition

**Fig. 1:**
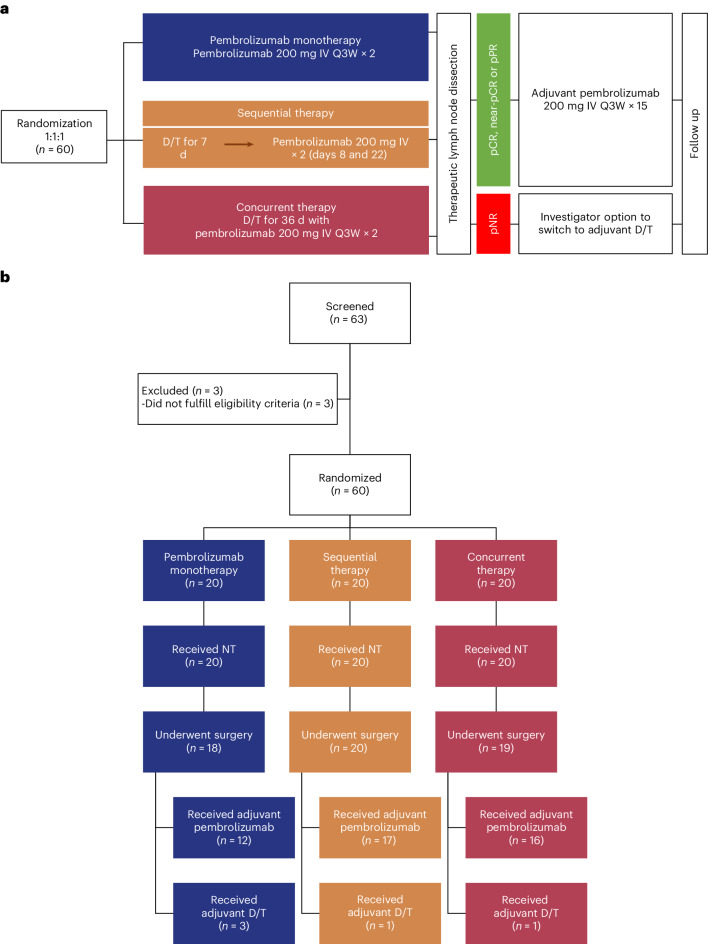
Trial design and patient flow diagram. **a**, Overview of the non-comparative randomized phase 2, open-label, three-arm NeoTrio trial (ClinicalTrials.gov registration: NCT02858921), with pathological response-directed adjuvant therapy. Dosing regimen for D/T was dabrafenib 150 mg PO BID plus trametinib 2 mg PO QD. **b**, Patient flow diagram. BID, twice per day; D/T, dabrafenib plus trametinib; NT, neoadjuvant therapy; PO, per oral; QD, once per day; Q3W, every three weeks.

Between 8 November 2017 and 18 May 2021, 63 patients with resectable *BRAF*^V600^-mutant stage III melanoma were screened, of which 60 were enrolled. Patients were randomized to treatment with 6 weeks of neoadjuvant pembrolizumab monotherapy (*n* = 20), sequential therapy (1 week of dabrafenib plus trametinib followed by pembrolizumab; *n* = 20) or concurrent triple therapy (pembrolizumab with dabrafenib plus trametinib; *n* = 20; Fig. 1). At baseline, the median age was 53 (interquartile range (IQR) = 42–63), 25 (42%) patients were females and all patients had confirmed *BRAF*^V600^ mutations; 49 (82%) patients had V600E, 9 (15%) had V600K and 2 (3%) had V600R (Table 1). Nodal basins included the axilla (43%), ilioinguinal (32%) and neck (32%). As per the American Joint Committee on Cancer (AJCC) eighth edition staging manual^28,29^, patients had either clinical N1b nodal disease (1 macroscopic node; 63%), N2b nodal disease (2–3 macroscopic nodes; 20%) or N3b nodal disease (≥4 macroscopic nodes or matted nodes; 17%).

**Table 1:** Patient disposition and baseline characteristics

Parameters	Pembrolizumab monotherapy (*n* = 20)	Sequential therapy (*n* = 20)	Concurrent therapy (*n* = 20)	Overall (*n* = 60)
Age in years, median (IQR)	56 (51, 64)	50 (38, 63)	53 (42, 61)	53 (42, 63)
Sex, *n* (%)				
Female	8 (40)	8 (40)	9 (45)	25 (42)
Male	12 (60)	12 (60)	11 (55)	35 (58)
ECOG status, *n* (%)				
0	20 (100)	19 (95)	20 (100)	59 (98)
1	0	1 (5)	0	1 (2)
*BRAF* mutation subtype, *n* (%)				
V600E	16 (80)	16 (80)	17 (85)	49 (82)
V600R	1 (5)	0	1 (5)	2 (3)
V600K	3 (15)	4 (20)	2 (10)	9 (15)
N category^a^, *n* (%)				
N1b	13 (65)	11 (55)	14 (70)	38 (63)
N2b	3 (15)	6 (30)	3 (15)	12 (20)
N3b	4 (20)	3 (15)	3 (15)	10 (17)
Nodal sites^b^, *n* (%)				
Axilla	7 (35)	9 (45)	10 (50)	26 (43)
Ilioinguinal	5 (25)	9 (45)	5 (25)	19 (32)
Neck	11 (55)	3 (15)	5 (25)	19 (32)

^a^AJCC eighth edition.

^b^Some patients had multiple nodal sites.

At data cut-off (26 October 2023), the median follow-up was 24.5 months (95% confidence interval (CI), 23.0–35.3). All 60 patients received at least one dose of neoadjuvant pembrolizumab and were included in the analysis. Of these, 51 (85%) patients completed neoadjuvant treatment and 9 (15%) patients discontinued early due to neoadjuvant treatment-related adverse events (TRAEs; 8 (40%) in the concurrent arm, none in the sequential arm and 1 (5%) in the pembrolizumab arm; Fig. 1 and Table 2). Following neoadjuvant treatment, 57 (95%) patients underwent surgery; 3 (5%) patients did not, due to distant progression. Following surgery, 45 (75%) patients received adjuvant pembrolizumab, 5 (8%) received adjuvant dabrafenib plus trametinib per investigator decision following pathological non-response (pNR) and 7 (12%) received no adjuvant therapy, either due to toxicity (*n* = 5), progression (*n* = 1) or poor quality of life following surgery due to pre-existing paraplegia (*n* = 1; Fig. 1).

**Table 2:** Summary of adverse events, interruptions and discontinuations relating to treatment

Parameters	Pembrolizumab monotherapy (*n* = 20)	Sequential therapy (*n* = 20)	Concurrent therapy (*n* = 20)
All TRAE, *n* (%)	17 (85)	19 (95)	20 (100)
Grade 3/grade 4 TRAE, *n* (%)	1 (5)	5 (25)	11 (55)
Treatment-related death, *n* (%)	0	0	0
During neoadjuvant therapy, *n*	20	20	20
All TRAE, *n* (%)	15 (75)	16 (80)	20 (100)
Grade 3/grade 4 TRAE, *n* (%)	1 (5)	1 (5)	8 (40)
Treatment discontinuation due to TRAE^a^	0	0	7 (35)
Dabrafenib plus trametinib	–	0	7
Pembrolizumab	0	0	1
Treatment interruption due to TRAE^a^	0	3 (15)	19 (95)
Dabrafenib plus trametinib	–	3	19
Pembrolizumab	0	0	0
During adjuvant pembrolizumab, *n*	12	17	16
Treatment discontinuation due to TRAE^b^	1	4	4
During adjuvant dabrafenib plus trametinib, *n*^c^	3	1	1
Treatment discontinuation due to TRAE	1	0	0

Summary of TRAEs, treatment interruptions and treatment discontinuations during neoadjuvant and adjuvant therapy.

^a^All interruptions and discontinuations during neoadjuvant therapy were due to TRAE.

^b^Six patients discontinued adjuvant pembrolizumab for reasons other than TRAE (recurrence, *n* = 6).

^c^Dabrafenib plus trametinib could be given as adjuvant therapy at the investigator’s discretion in any patient with a pNR.

### Efficacy: response

Pathological, radiological and metabolic response rates are shown in Table 3. The primary endpoint was met in all three arms (pathological response rate of ≥5%). A pathological response was achieved in 55% (11/20; 95% CI, 36–83) of patients in the pembrolizumab arm, 50% (10/20; 95% CI, 27–73) in the sequential arm and 80% (16/20; 95% CI, 60–97) in the concurrent arm; a pCR was reported in 30% (6/20), 15% (3/20) and 50% (10/20) of patients, respectively (Table 3). A major pathological response (MPR; pCR or near-pCR) occurred in 40% (8/20) of patients with pembrolizumab monotherapy, 30% (6/20) of patients with sequential therapy and 55% (11/20) of patients with concurrent therapy. A pNR occurred in 35% (7/20) of patients treated with pembrolizumab, 50% (10/20) of patients with sequential therapy and 15% (3/20) of patients with concurrent therapy.

**Table 3:** Pathological, radiological and metabolic responses at week 6

Parameters	Pembrolizumab monotherapy (*n* = 20)	Sequential therapy (*n* = 20)	Concurrent therapy (*n* = 20)
Pathological response, *n* (%) (95% CI)	11 (55) (36, 83)	10 (50) (27, 73)	16 (80) (60, 97)
MPR	8 (40)	6 (30)	11 (55)
pCR	6 (30)	3 (15)	10 (50)
Near-pCR	2 (10)	3 (15)	1 (5)
pPR	3 (15)	4 (20)	5 (25)
pNR	7 (35)	10 (50)	3 (15)
NE^a^	2 (10)	0	1 (5)
Radiological response,^b^ *n* (%) (95% CI)	6 (30) (11, 54)	10 (50) (23, 68)	14 (70) (46, 88)
CR	3 (15)	0	7 (35)
PR	3 (25)	10 (50)	7 (35)
SD	9 (45)	9 (45)	5 (25)
PD	5 (25)	1 (5)	1 (5)
NE	0	0	0
Metabolic response,^c^ *n* (%)	8 (40)	10 (50)	16 (80)
CMR	1 (5)	1 (5)	3 (15)
PMR	7 (35)	9 (45)	13 (65)
SMD	4 (20)	7 (35)	0
PMD	4 (20)	3 (15)	1 (5)
NE^d^	4 (20)	0	3 (15)

^a^Three patients did not undergo surgery due to metastatic progression during neoadjuvant therapy.

^b^Objective response per RECIST version 1.1.

^c^Objective response per EORTC PET criteria.

^d^Seven patients had missing or incomplete PET data.

NE, non-evaluable; SMD, stable metabolic disease.

**Extended Data Fig. 1:**
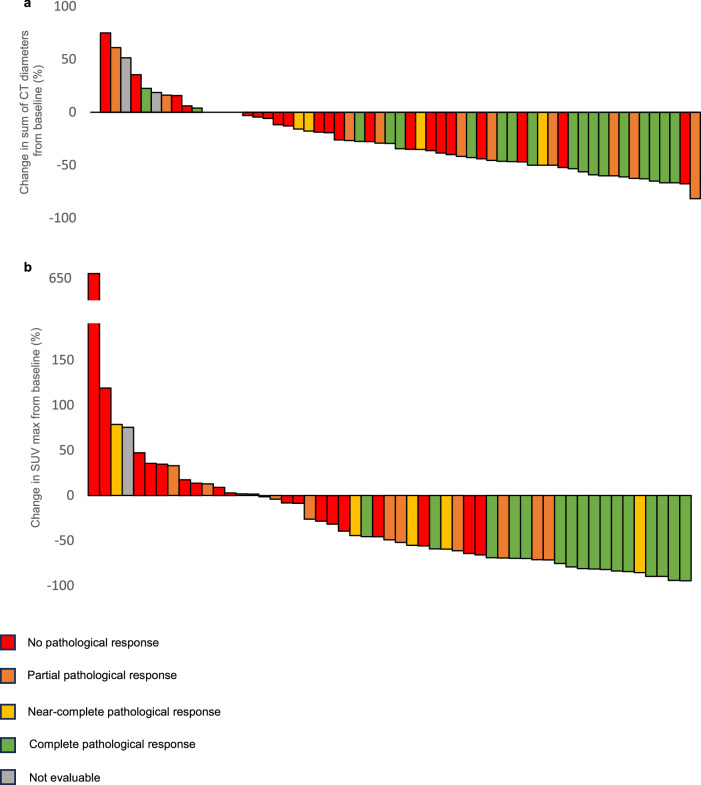
Waterfall plots of percentage change in tumor burden following neoadjuvant therapy by pathological response. **a**, Percentage change from baseline in sum of CT target lesions. **b**, Percentage change from baseline in SUVmax. CT, computed tomography; SUV, standardized uptake value.

An objective radiological response (CR or PR) per Response Evaluation Criteria in Solid Tumors (RECIST) version 1.1 (ref. ^30^) was observed in 30% (6/20) of patients treated with pembrolizumab, 50% (10/20) of patients treated with sequential treatment and 70% (14/20) of patients treated with concurrent treatment (Table 3). There were seven (12%) patients with RECIST progressive disease (PD; three distant and four locoregional lymph nodes; 5/20 patients (25%) in the pembrolizumab arm, 1/20 (5%) in the sequential arm and 1/20 (5%) in the concurrent arm). An MPR was found in 70% (7/10) of patients with RECIST CR, 55% (11/20) of patients with RECIST PR, 30% (7/23) of patients with RECIST stable disease (SD) and no patient with RECIST PD (Extended Data Table 1 and Extended Data Fig. 1). A pNR was found in 30% (6/20) and 52% (12/23) of patients with RECIST PR and SD, respectively. RECIST CR selected patients with an MPR with poor sensitivity (36%) and high specificity (92%). Similarly, RECIST PD selected patients with pNR with poor sensitivity (10%) and high specificity (95%).

A metabolic response (complete metabolic response (CMR) or partial metabolic response (PMR)) per the European Organization for Research and Treatment of Cancer (EORTC) positron emission tomography (PET) criteria^31^ was observed in 40% (8/20) of patients treated with pembrolizumab, 50% (10/20) of those treated with sequential therapy and 95% (19/20) of those treated with concurrent therapy, including five CMR in the concurrent arm and one CMR in each of the sequential and pembrolizumab monotherapy arms (Table 3). An MPR was found in 57% (4/7 had pCR) of patients with a CMR, 60% (18/30) of patients with a PMR, but no patient with stable metabolic disease and only two patients (25%) with progressive metabolic disease (PMD; Extended Data Table 2 and Extended Data Fig. 1). pNR was found in 23% (7/30) and 78% (7/9) of patients with partial or stable metabolic response, respectively. CMR selected patients with an MPR with poor sensitivity (22%) and high specificity (92%). Similarly, PMD selected patients with pNR with poor sensitivity (25%) and high specificity (91%).

### Efficacy: recurrence and survival

At data cut-off, there were 18 patients (30%) with postsurgical recurrence, and nine (15%) patients had died. All deaths followed presurgical progression (2/9) or postsurgical recurrence (7/9) events, and all were attributed to melanoma.

**Fig. 2:**
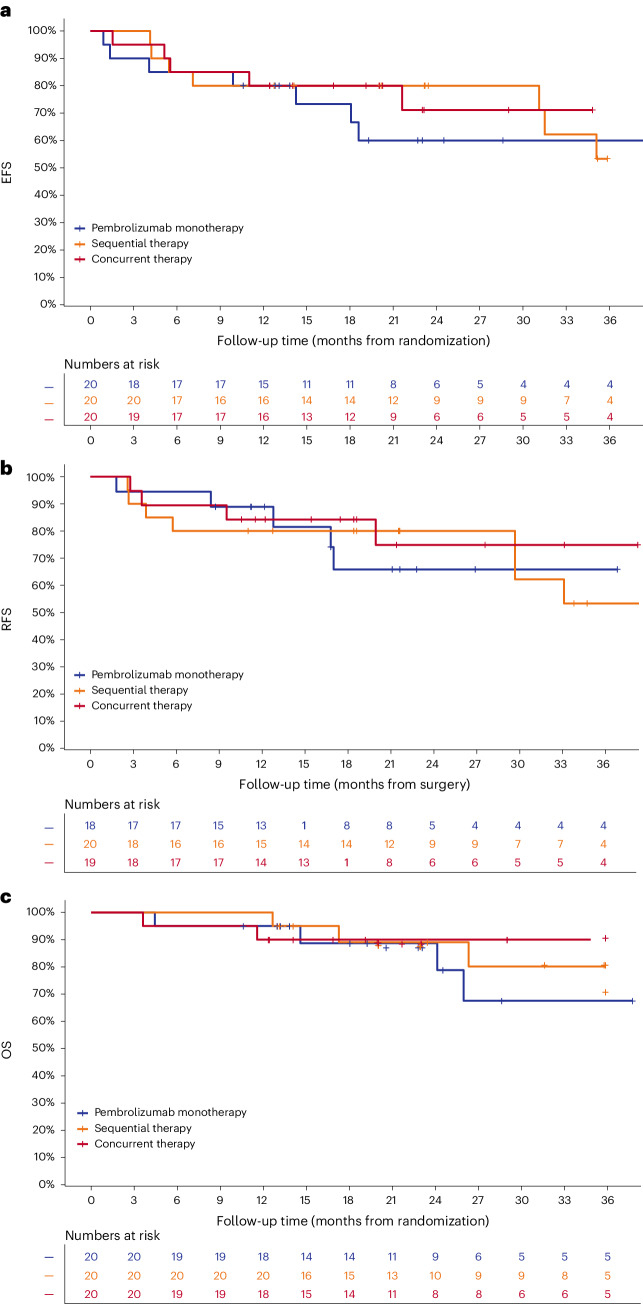
Kaplan–Meier survival estimates per treatment arm. Landmark survival rates were calculated at 12 and 24 months. **a**, EFS curve showing 12-month EFS rate was 80% in all arms; 24-month EFS was 60% with pembrolizumab monotherapy, 80% with sequential therapy and 71% with concurrent therapy. **b**, RFS curve showing 12-month RFS rate was 89% with pembrolizumab monotherapy, 80% with sequential therapy and 84% with concurrent therapy; 24-month RFS rate was 66%, 80% and 75%, respectively. **c**, OS curve showing 12-month OS rate was 94% with pembrolizumab monotherapy, 95% with sequential therapy and 95% with concurrent therapy; 24-month OS rate was 76%, 89% and 95%, respectively.

At data cut-off, there were nine (45%), nine (45%) and six (30%) defined events for the EFS in the pembrolizumab alone, sequential and concurrent arms, respectively (Table 4). The landmark EFS rates at 12 and 24 months were 80% and 60% with pembrolizumab alone, 80% and 80% with sequential treatment and 80% and 71% with concurrent treatment (Fig. 2 and Table 4).

**Table 4:** Median and landmark survival outcomes

Parameters	Pembrolizumab monotherapy (*n* = 20)	Sequential therapy (*n* = 20)	Concurrent therapy (*n* = 20)
EFS			
Events to data cutt-off, *n*^a^	7	8	5
Median EFS, months	NR	NR	NR
12-month			
EFS events, *n*	4	4	4
EFS rate, % (95% CI)	80 (64, 100)	80 (64, 100)	80 (64, 100)
24-month			
EFS events, *n*	7	4	5
EFS rate, % (95% CI)	60 (40, 89)	80 (64, 100)	71 (52, 98)
RFS			
Events to data cut-off, *n*^b^	5	4	8
Median RFS, months	NR	NR	NR
12-month			
RFS events, *n*	2	4	3
RFS rate, % (95% CI)	89 (75, 100)	80 (64, 100)	84 (69, 100)
24-month			
RFS events, *n*	5	4	4
RFS rate, % (95% CI)	66 (45, 96)	80 (64, 100)	75 (55, 100)
OS			
Deaths to data cut-off, *n*	3	3	1
Median OS, months	NR	NR	NR
12-month			
OS events, *n*	1	1	1
OS rate, % (95% CI)	94 (84, 100)	95 (85, 100)	95 (85, 100)
24-month			
OS events, *n*	3	2	1
OS rate, % (95% CI)	76 (55, 100)	89 (75, 100)	95 (85, 100)

EFS, RFS and OS event numbers and landmark rates.

^a^EFS events include presurgical progression, postsurgical recurrence or death.

^b^RFS events include postsurgical recurrence or death.

NR, not reached.

At data cut-off, there were six (30%), eight (40%) and five (25%) recurrence events or deaths in the pembrolizumab alone, sequential and concurrent arms, respectively (Table 4). The landmark RFS rates at 12 and 24 months were 89% and 66% with pembrolizumab alone, 80% and 80% with sequential treatment and 84% and 75% with concurrent treatment (Fig. 2 and Table 4).

**Extended Data Fig. 2:**
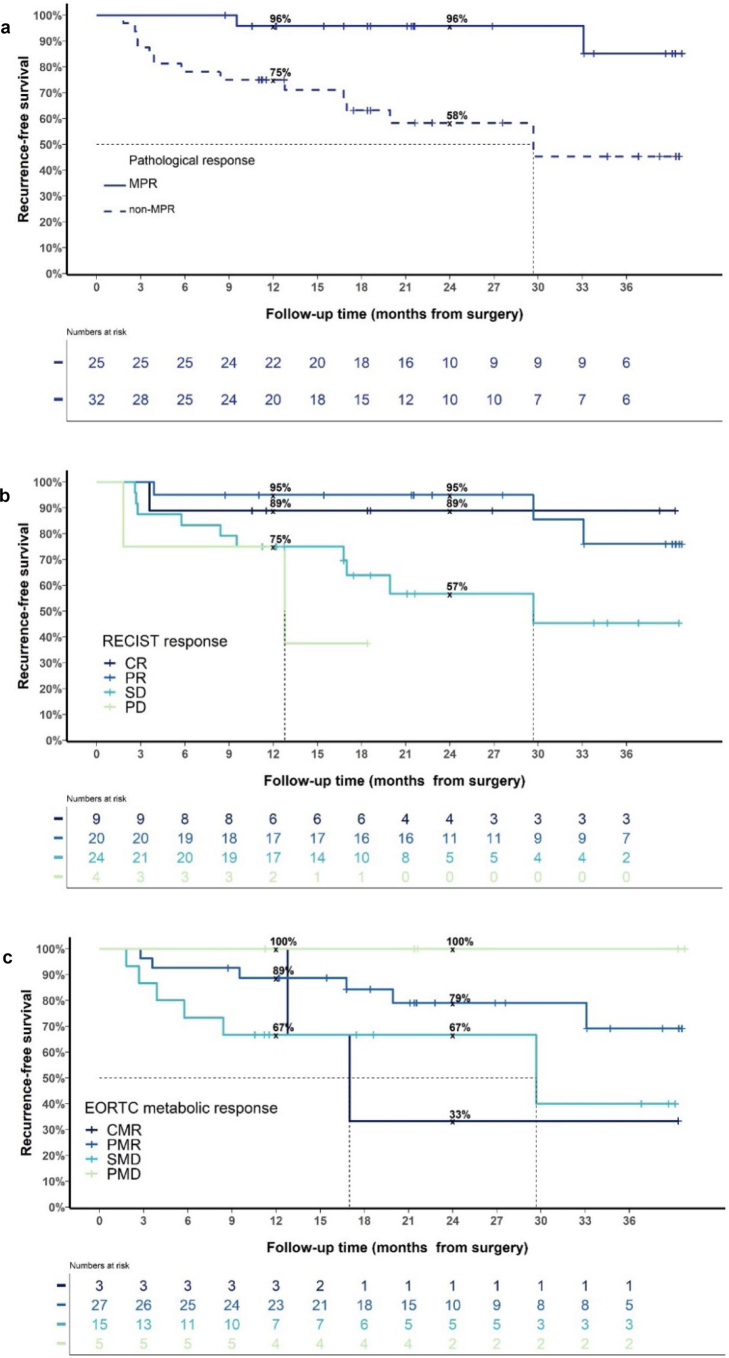
Recurrence-free survival by response outcomes. Recurrence-free survival in all patients who underwent surgery by (**a**) pathological response, (**b**) radiographic (RECIST v1.1) response and (**c**) metabolic (EORTC PET) response. CMR, complete metabolic response; CR, complete response; MPR, major pathological response; PD, progressive disease; PMD, progressive metabolic disease; PMR, partial metabolic response; PR, partial response; RFS, recurrence-free survival; SD, stable disease; SMD, stable metabolic disease.

**Extended Data Fig. 3:**
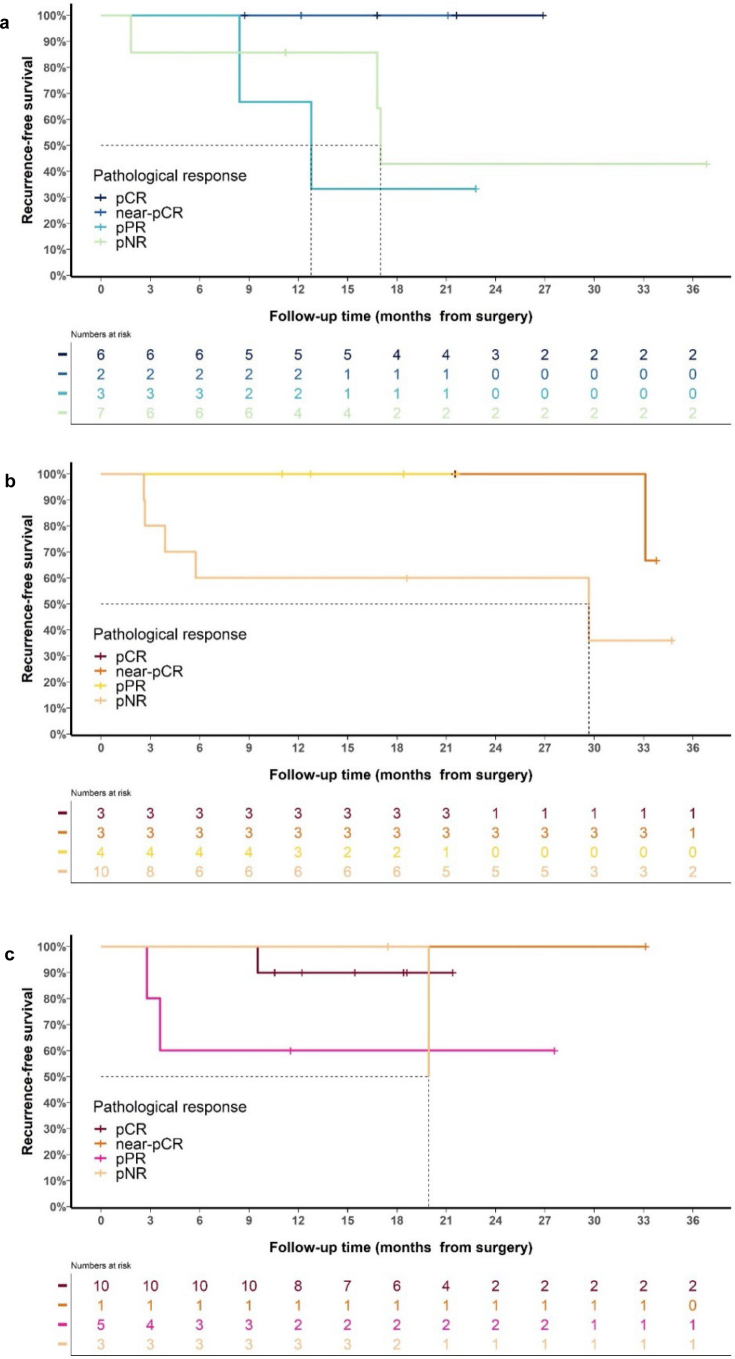
Recurrence-free survival by pathological response. Recurrence-free survival by pathological response with (**a**) pembrolizumab monotherapy, (**b**) sequential therapy and (**c**) concurrent therapy. MPR, major pathological response; pCR, pathological complete response; pNR, pathological non-response; pPR, pathological partial response; RFS, recurrence-free survival.

The RFS rate was higher in patients with MPR versus those without MPR (Extended Data Figs. 2 and 3 and Extended Data Table 3). In the pembrolizumab monotherapy arm, no patient with MPR recurred. One patient with MPR recurred in each of the targeted therapy arms (a patient with near-pCR in the sequential therapy arm who recurred >2 years after surgery and a patient with pCR in the concurrent arm who recurred 10 months after surgery). Pathological response was a better predictor of RFS than imaging response measures, with landmark RFS rates at 12 and 24 months of—96% and 96% for MPR; 89% and 89%, respectively, for RECIST CR; 95% and 95% for RECIST PR; 100% and 33% for EORTC CMR; and 89% and 79% for EORTC PMR.

At data cut-off, there were four (20%), three (15%) and two (10%) deaths in the pembrolizumab alone, sequential and concurrent arms, respectively (Table 4). The landmark OS rates at 12 and 24 months were 95% and 76% with pembrolizumab alone, 95% and 89% with sequential treatment and 95% and 95% with concurrent treatment (Fig. 2 and Table 4).

### Adverse events

Overall, TRAEs of any grade occurred in 85% (17/20) of patients treated with pembrolizumab monotherapy, 95% (19/20) of patients with sequential therapy and 100% (20/20) of patients with concurrent therapy (Extended Data Table 4). Most TRAEs occurred during neoadjuvant therapy; 75% (15/20) of patients were treated with pembrolizumab monotherapy, 80% (16/20) of patients were treated with sequential therapy and 100% (20/20) of patients were treated with concurrent therapy. Overall, grade 3/grade 4 TRAEs occurred in 5% (1), 25% (5) and 55% (11) of patients, respectively, with grade 3/grade 4 TRAEs during neoadjuvant therapy occurring only in the concurrent arm (40%; 8/20; Table 2). Pyrexia was the most common TRAE, occurring in 21 patients (17 in the concurrent arm), followed by fatigue, rash, pruritis, vitiligo, headache and nausea (Extended Data Table 4).

Twenty-two patients had interruptions to neoadjuvant dabrafenib/trametinib treatment due to TRAEs (19 (95%) in the concurrent arm and 3 (15%) in the sequential arm; Table 2); there were no interruptions to neoadjuvant pembrolizumab. Seven patients discontinued neoadjuvant therapy early due to TRAEs, all from the concurrent arm (six of whom ceased dabrafenib/trametinib and one who ceased both dabrafenib/trametinib and pembrolizumab). Four of the seven patients had ≥1 TRAE. TRAEs leading to early discontinuation of dabrafenib/trametinib ranged from grades 1–3 and included pyrexia (*n* = 3), chills (*n* = 2) and elevated alanine transferase and aspartate transferase (*n* = 1). The sole patient who ceased neoadjuvant pembrolizumab had grade 3 hepatitis and grade 3 drug-induced liver injury attributed to pembrolizumab, resulting in the cessation of all three agents. All patients who ceased neoadjuvant treatment fully recovered from toxicity. At data cut-off, two of the seven patients had recurred (one with pCR who recurred in the liver and one with pNR who recurred in the spleen), and both were on survival follow-up.

Twenty-six (59%) patients completed all planned doses of adjuvant pembrolizumab. TRAEs were the primary reason for early discontinuation of adjuvant pembrolizumab (one (5%) patient in the pembrolizumab arm, four (20%) in the sequential arm and four (30%) in the concurrent arm; Table 2). In the six patients who received adjuvant dabrafenib plus trametinib, five completed 12 months of treatment as planned and one received 9 months of treatment with discontinuation due to peripheral neuropathy.

Surgical complications occurred in 39 patients overall (68%; Extended Data Table 5). There were nine grade 3/grade 4 surgical adverse events (four with pembrolizumab monotherapy, three with sequential therapy and two with concurrent therapy), including five cases of wound infection requiring hospitalization.

The ease of upfront resection (estimated at baseline) was compared with the ease of resection after 6 weeks of neoadjuvant therapy (recorded within 24 h after surgery; Extended Data Table 6). There were ten patients for whom resection was more difficult than estimated at baseline, due to the presence of inflammatory tissue or immunotherapy-related changes in four of six patients for whom reasons were provided. Neoadjuvant therapy was associated with improved resectability in eight (47%) patients in the concurrent arm, four (21%) patients in the sequential arm and two (12%) patients in the pembrolizumab arm. For most patients (55%), there was no difference, although resectability was harder in five (29%) patients in the pembrolizumab arm (two with pNR and three with MPR). When analyzed by lymph node basin or pathological response, all eight patients with improved resectability in the concurrent arm had pCR or pPR; however, overall, there was no difference when assessed by the pathological response. Resections were harder in neck nodal basin resections (27% (4/15)) compared with axillary (14% (3/22)) and ilioinguinal (18% (3/17)) resections.

### Protocol amendment

Eleven patients were enrolled before the protocol amendment, resulting in surgery at 7–12 weeks for ten patients (four from the pembrolizumab monotherapy arm and three each from the sequential and concurrent arms) rather than at 6 weeks. There was no increased incidence of presurgical progression, recurrence, death or toxicity events in this group.

### Health-related quality of life (HRQOL)

**Extended Data Fig. 4:**
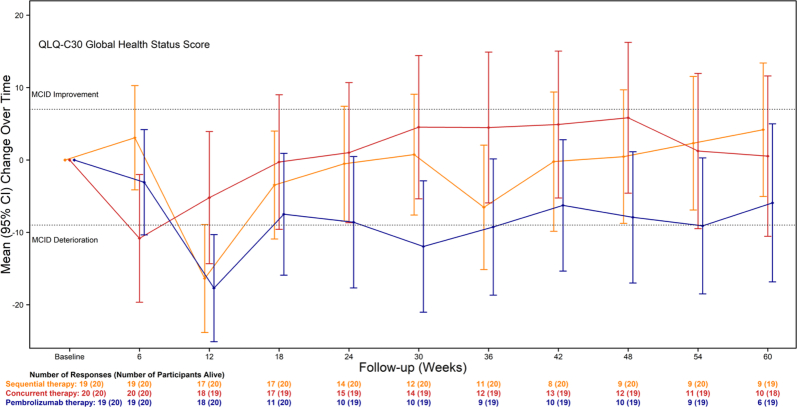
Change in EORTC QLQ-C30 Global Health Score over time. Patients were surveyed at 6-week intervals from baseline (censored at week 60 due to small number of responses (<5)). Data are presented as mean values ± 95% CI. Mean change from baseline was compared to established levels of MCID1 improvement or deterioration. A number of responses (*n*) at each timepoint are displayed in the data table (range per treatment arm, 6–20). CI, confidence interval; EORTC QLQ-C30, European Organization for Research and Treatment of Cancer’s Core Quality of Life Questionnaire; MCID, minimum clinically important difference.

Statistically significant deterioration in HRQOL was only reported in the perioperative period—week 6 (presurgery) for patients treated with concurrent therapy (Global Health Score (GHS) mean change: −10.83 (95% CI, −19.63 to −2.01), *P* < 0.05) and at week 12 (postsurgery) for patients treated with either pembrolizumab monotherapy (GHS mean change: −17.70 (95% CI, −25.10 to −10.30), *P* < 0.001) or sequential therapy (GHS mean change: −16.37 (95% CI, −23.83 to −8.92), *P* < 0.001; Extended Data Fig. 4). Patients treated with pembrolizumab monotherapy also had a statistically significant decline at week 30 (mean change: −11.95 (95% CI, −21.02 to −2.87), *P* < 0.05). However, the only deterioration deemed to be clinically significant was in the pembrolizumab monotherapy arm at week 12.

## Discussion

The NeoTrio trial investigated combination BRAF-targeted therapy and ICI in the neoadjuvant setting for resectable melanoma, including an anti-PD-1 monotherapy comparator arm. All regimens elicited a substantial pathological response rate (≥50% of patients) and met the primary endpoint. Concurrent therapy induced the highest pathological response rate, similar to that observed in previous neoadjuvant studies of BRAF-targeted therapy alone (67%)^25,26^, but the durability is uncertain. Across all arms, patients with MPR had fewer recurrences than those without MPR; however, no patient with MPR in the pembrolizumab arm has recurred, compared with one each from the arms containing targeted therapy. Concurrent therapy was the most toxic, with 55% of patients experiencing grade 3/grade 4 adverse events and a 40% discontinuation rate during neoadjuvant therapy. This mirrors experience in the advanced setting, where triple therapy has induced high rates of grade 3/grade 4 adverse events, ranging from 55% to 79%^14–16^. Overall, pending longer survival follow-up, we do not endorse the addition of BRAF-targeted therapy to neoadjuvant ICI in melanoma.

In the NeoTrio trial, it was hypothesized that response to neoadjuvant anti-PD-1 treatment would be improved by a short course of BRAF/MEK inhibitor induction therapy. However, the pathological response rate with induction therapy in the sequential arm (50% (95% CI, 27–73)) was similar to pembrolizumab alone (55% (95% CI, 36–83)). Administering BRAF/MEK inhibitors and ICI in sequence has been previously examined in the metastatic setting. In SECOMBIT, 8 weeks of encorafenib plus binimetinib followed by nivolumab plus ipilimumab (and further encorafenib plus binimetinib if progression) was no more effective than nivolumab plus ipilimumab (and further encorafenib plus binimetinib if progression)^17^. This suggests that the T cells induced by BRAF/MEK inhibition in melanoma tumors are not conducive to a heightened anti-PD-1 response and may not be tumor-specific but rather a non-specific immune response to cell death induced by the BRAF-targeted therapy. Potential mechanisms of cross-resistance between the two treatment modalities have been explored, with several common pathways identified including mitogen-activated protein kinase (MAPK)^32^ and PTEN^33^. Further investigation is essential to understand the lack of synergy, and the translational analysis of tissue samples from NeoTrio is ongoing^34^.

Although the response rate was highest with concurrent therapy, the quality and duration of response must be observed carefully. In multiple settings for *BRAF*-mutant melanoma, targeted therapy has been associated with a good initial response rate, but a higher risk of treatment failure when compared with patients who respond to immunotherapy. In a pooled analysis, 37% of patients had a pCR to neoadjuvant immunotherapy. Two-year RFS for complete responders was excellent, at 96%; however, any pathological response was associated with excellent durability (for example, 2-year RFS of 94% for partial responders). In contrast, 47% of patients had a pCR to targeted therapy, but this afforded a substantially lower 2-year RFS of 79%; partial responders had a 2-year RFS of 18%^25^. Recurrences with targeted therapy tended to occur late (>12–18 months)^25^, but still within 2 years^26^. With a median follow-up of 24.5 months, the only recurrences in MPR patients in NeoTrio have occurred in targeted therapy arms. Long-term follow-up is required to monitor for further late recurrences in each of the arms. Despite the higher response rate, loss of prognostic certainty is an important consideration when balancing the advantages and disadvantages of adding targeted therapy to anti-PD-1.

There is a strong correlation between pathological response to neoadjuvant ICI and long-term survival^25^. In an updated analysis of the OpACIN and OpACIN-neo trials, 3-year RFS was 95% for patients with any pathological response versus 37% with pNR (*P* < 0.001)^35^. In a recent trial of neoadjuvant nivolumab plus relatlimab, 2-year RFS was 92% in patients with a pathological response and 55% with non-response (*P* = 0.005)^36^. Importantly, previous neoadjuvant trials have reported that pathological response outcomes are often better than anticipated by presurgical RECIST response^22,25,36,37^. In the NeoTrio trial, almost half (46%) of patients with no objective radiological response had a pathological response, including seven with MPR. Interestingly, fluorodeoxyglucose (FDG)-PET CMR was more specific for MPR than RECIST CR, yet both modalities had poor sensitivity. Pathological response is currently the best surrogate marker for survival following neoadjuvant therapy^25,35^, and our data showed that pathological response at week 6 predicted RFS better than RECIST or metabolic response at week 6. More data are required to evaluate the relationship between pathological response and metabolic response.

Around 35% of patients do not have a pathological response to neoadjuvant immunotherapy (pooled anti-PD-1 monotherapy and anti-PD-1 plus anti-CTLA-4) and are at high risk of postsurgical recurrence, with a 2-year RFS of 37%^25^. Future studies should focus on new immune approaches that target non-responders to provide a durable response. Pathological response to anti-PD-1, with and without anti-CTLA-4, has been shown to correlate with high interferon-γ expression and high tumor mutation burden, suggesting biomarker-driven treatment investigation may be appropriate^38,39^. The neoadjuvant platform is an ideal vehicle for testing new approaches due to the rapid readout and correlation of pathological responses with survival outcomes that allow quick and definitive ‘go’ or ‘no-go’ decisions on new agents, combinations or regimens. For example, in the DONIMI trial, a theoretically promising combination of the HDAC inhibitor domatinostat and anti-PD-1 was quickly discarded when 6 weeks of neoadjuvant combination therapy provided no pathological response benefit (and possibly a measurable detriment) compared with anti-PD-1 alone^38^. Investigation of alternative immune approaches in the neoadjuvant setting is ongoing, including with anti-LAG-3 antibody relatlimab, which was recently found to provide a 70% pathological response rate when combined with nivolumab^36^. Combination treatment with drugs such as Janus kinase (JAK) inhibitors to target therapeutic resistance to immunotherapy may also be explored^40^.

HRQOL was lowest following neoadjuvant treatment in the concurrent arm (week 6) and following surgery in the pembrolizumab monotherapy and sequential arms (week 12). High rates of toxicity in the concurrent arm correlated with a deterioration in HRQOL at week 6 before surgery. Substantial decrements in HRQOL were observed in the postoperative period with sequential and pembrolizumab monotherapy, but not with concurrent therapy, and can be attributed to a lower rate of surgical adverse events in the concurrent arm. Interestingly, resectability was more frequently assessed as being ‘easier’ compared with surgical expectations at baseline in the concurrent arm, and the pathological response rate was higher, which may explain the maintenance of HRQOL from weeks 6 through 12. Further analyses and correlation at the patient-specific level regarding drug and surgical adverse events are ongoing.

The study was limited in several ways. The NeoTrio trial was not powered to make statistical comparisons between the three arms, which limits the interpretation of the findings. The small sample size also makes it difficult for clear trends to emerge in long-term survival and HRQOL.

To conclude, the NeoTrio trial suggests that BRAF-targeted therapy should not be added to neoadjuvant ICI for *BRAF*^V600^-mutant patients, at risk of higher toxicity and loss of curative potential. Longer-term follow-up will be critical to determining the durability and quality of response, particularly in the targeted therapy arms.

## Methods

### Patients and study design

NeoTrio (ClinicalTrials.gov registration: NCT02858921) was a randomized, multi-arm, open-label, parallel phase II study of neoadjuvant pembrolizumab, dabrafenib and trametinib in patients with *BRAF*^V600^-mutant resectable stage III cutaneous or unknown primary melanoma. Patients were ≥18 years of age with histologically confirmed resectable AJCC eighth edition^29^ stage IIIB–D melanoma with nodal disease (no in-transits) and sufficient tissue to enable multiple core and excision biopsies. Unresected primary melanoma was allowed. Patients had measurable disease per RECIST version 1.1 (ref. ^30^; ≥15 mm shortest diameter for lymph nodes), *BRAF*^V600^ mutation positivity, adequate organ function and Eastern Cooperative Oncology Group (ECOG) performance status ≤1. Patients were enrolled at three sites in Australia (Westmead Hospital, Melanoma Institute Australia and Peter MacCallum Cancer Center). The study was performed in accordance with the Declaration of Helsinki and Good Clinical Practice guidelines. Patients provided written informed consent. The study protocol was approved by the human research ethics committee at each participating institution. Key sections of the study protocol are given in Supplementary Information.

### Treatment

Patients received neoadjuvant therapy for 6 weeks across the three parallel treatment arms. Patients were randomized in a 1:1:1 ratio via a web-based system in permuted blocks (block sizes 6 and 9) and stratified by *BRAF*^V600E^ versus non-*BRAF*^V600E^ (that is, V600K, V600R, V600D and V600M) mutation. Patients allocated to pembrolizumab monotherapy received pembrolizumab 200 mg intravenously (IV) on days 1 and 22. Patients allocated to sequential therapy received targeted therapy (dabrafenib 150 mg orally twice per day and trametinib 2 mg orally once per day) for 7 days, followed by pembrolizumab 200 mg IV on days 8 and 22. Patients allocated to concurrent therapy received targeted therapy as above for 6 weeks, with pembrolizumab 200 mg IV on days 1 and 22. Following neoadjuvant therapy, all patients underwent therapeutic lymph node dissection (between days 42 and 56). After surgery, patients received 42 weeks (16 doses) of adjuvant pembrolizumab administered every 3 weeks, with the first adjuvant dose given as soon as possible after surgery, until toxicity, withdrawn consent, disease recurrence or death. For patients with pNR to neoadjuvant therapy, investigators/patients had the choice to switch from adjuvant pembrolizumab to standard-of-care dabrafenib and trametinib outside of the protocol for 52 weeks duration.

### Assessments and outcomes

Self-reported biological sex was recorded. *BRAF*^V600^ mutation status was confirmed before study enrollment by immunohistochemistry or local molecular testing (for example, Oncofocus). At baseline, all patients underwent computed tomography (CT), magnetic resonance imaging and FDG-PET scans. CT scans were conducted every 6 weeks until the surgery, and then every 12 weeks afterward. FDG-PET scans were repeated at weeks 6 and 48. Tumor samples were collected at baseline, day 8 and day 15, followed by complete resection at week 6. A proportion of patients underwent surgery at week 12, before a protocol amendment to align with International Neoadjuvant Melanoma Consortium (INMC) guidelines^41^. Samples of peripheral blood for biomarker analysis were collected at baseline, day 8, day 15 and day 42 (before surgery) and at disease progression or recurrence, if applicable. Stool samples for microbiome analysis were collected at baseline, within 2 weeks before surgery, week 24 and at disease progression or recurrence, if applicable. HRQOL was assessed every 6 weeks using the EORTC Quality of Life Questionnaire-C30 (ref. ^42^) and after surgery using FACT-M^43^ (questions M10–M17). Safety was monitored continuously throughout the trial. Surgical outcomes were assessed for 4 weeks after surgery. Resectability, as per INMC guidelines,^44^ was assessed at baseline and week 6 (within 24 h after surgery). Survival follow-up continued every 12 weeks for up to 10 years.

The primary endpoint was pathological response rate and pCR rate based on examination of the resected tumor at week 6 by central pathologist review (R.A.S.). Definitions of pathological response categories per the INMC^45^ are given in Supplementary Table 1 and Supplementary Information. Secondary endpoints included radiological response (RECIST version 1.1 (ref. ^30^)) at week 6 before surgery, RFS (defined as the time from surgery to recurrence or death), OS, surgical outcomes, safety, HRQOL and translational endpoints comparing immunological, proteomic and genetic biomarkers in tumor tissue and blood samples. Other endpoints were metabolic response (EORTC PET^31^) at week 6 before surgery and EFS (defined as the time from treatment start to progression, recurrence, or death).

### Statistical methods

Each arm was designed as a single-arm study. The sample size of 20 patients per arm (*n* = 60) was calculated to determine whether the pathological response rate was ≤5% or ≥20%. If the number of pathological responses was three or more, the hypothesis that a pathological response rate ≤5% was rejected with a target error rate of 0.080 and an actual error rate of 0.075. If the number of pathological responses was two or less, the hypothesis that a pathological response rate ≥20% was rejected with a target error rate of 0.210 and an actual error rate of 0.206 (refs. ^46,47^). With the exception of HRQOL, outcomes were measured in the intention-to-treat population, which included all patients who received at least one dose of pembrolizumab. Patients who dropped out before the first dose were replaced, except in the case of disease progression or death.

Patient characteristics are summarized by arm using descriptive statistics. The primary and secondary response outcomes were summarized using frequency and proportion by arm along with the two-sided 95% Clopper–Pearson exact CIs. The secondary time-to-event outcomes (EFS, RFS and OS) were analyzed using the Kaplan–Meier method stratified by arm. Twelve-month landmark rates for each survival outcome were provided for up to 3 years. RFS curves stratified by MPR (pCR or near-pCR response versus pPR or pNR) were drawn within each arm. No formal statistical inference to compare arms was computed.

HRQOL outcomes were assessed in all patients who completed baseline and ≥1 follow-up questionnaire. Data were censored at 60 weeks due to a small number (<5) of patients. Mean change over time with respect to baseline and 95% CI were calculated using mixed linear modeling including cohort, time (as a categorical variable) and a random intercept effect. A two-sided *P* ≤ 0.05 was considered statistically significant, with mean changes assessed with respect to established minimal clinically important difference values^48^. All statistical analyses were performed using SAS (version 9.4) and R (version 4.1.3).

### Reporting summary

Further information on research design is available in the Nature Portfolio Reporting Summary linked to this article.

## Online content

Any methods, additional references, Nature Portfolio reporting summaries, source data, extended data, supplementary information, acknowledgements, peer review information; details of author contributions and competing interests; and statements of data and code availability are available at 10.1038/s41591-024-03077-5.

## Supplementary information

Supplementary Information Supplementary Table 1 and Supplementary Note (NeoTrio Protocol V4.0 sections).

Reporting Summary

## Figures and Tables

**Extended Data Table 1:**
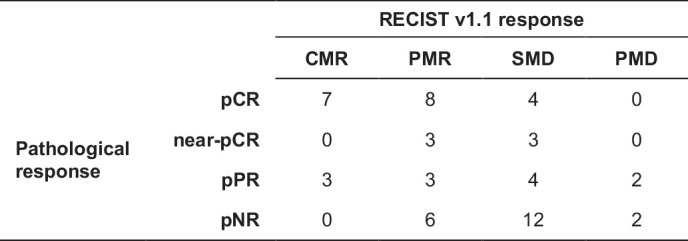
Concordance of pathological and radiographic response

All 57 patients who underwent surgery had both pathological and radiographic response data. CR, complete response; PD, progressive disease; pCR, pathological complete response; pNR, pathological non-response; pPR, pathological partial response; PR, partial response; RECIST, Response Evaluation Criteria in Solid Tumors; SD, stable disease.

**Extended Data Table 2:**
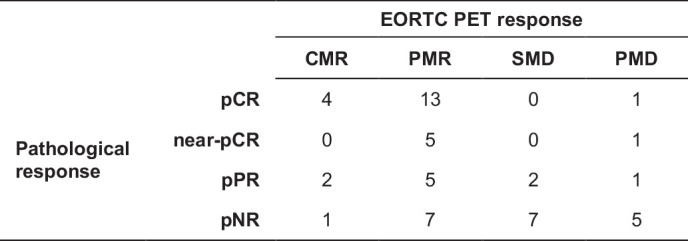
Concordance of pathological and metabolic response

Fifty-four patients had both pathological and metabolic response data. Six patients had only pathologic response data (no pre-surgical scan (*n* = 2), half-body scan at baseline (*n* = 1)) or only metabolic response data (no surgery due to distant progression (*n* = 3)). CMR, complete metabolic response; EORTC, European Organization for Research and Treatment of Cancer; PMD, progressive metabolic disease; pCR, pathological complete response; PET, positron emission tomography; pNR, pathological non-response; pPR, pathological partial response; PMR, partial metabolic response; SMD, stable metabolic disease.

**Extended Data Table 3:**
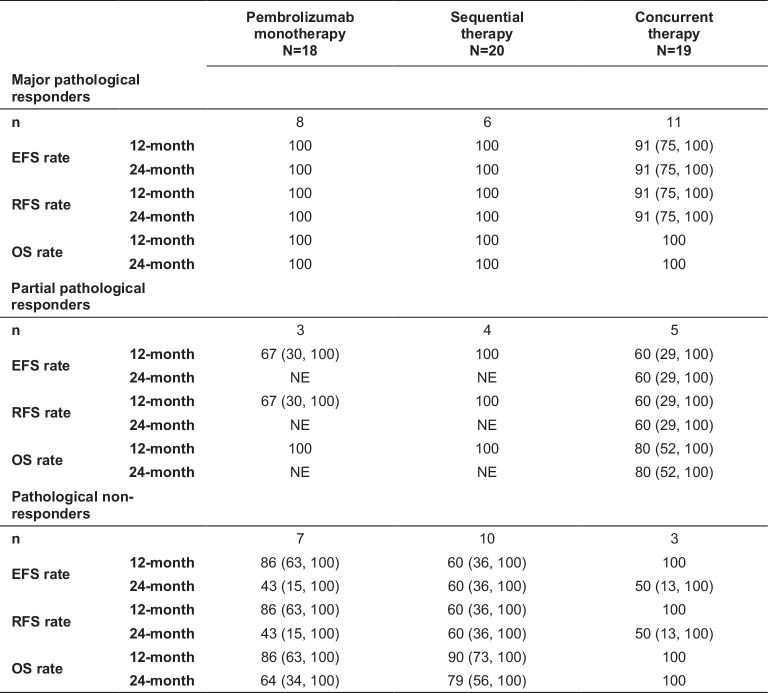
Survival rates by treatment arm and pathological response

Excludes the three patients who progressed to distant metastases during the neoadjuvant period.

Unless stated, values are % (95% CI). CI, confidence interval; EFS, event-free survival; NE, non-evaluable; OS, overall survival; RFS, recurrence-free survival.

**Extended Data Table 4:**
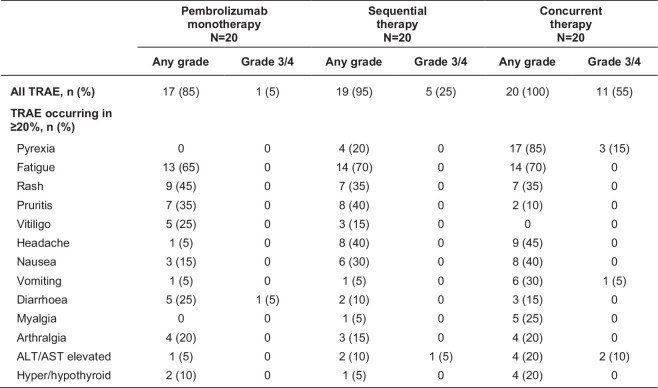
Treatment-related adverse events

Treatment-related adverse events occurring in ≥20% of patients at any stage during treatment. AE, adverse events; ALT, alanine transferase; AST, aspartate transferase; TRAE, treatment-related adverse event.

**Extended Data Table 5:**
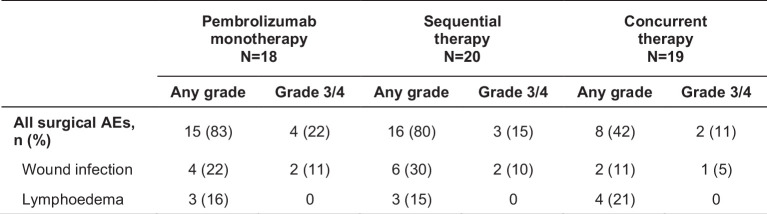
Surgery-related adverse events

Surgery-related adverse events were assessed in all 57 patients who underwent surgery. AE, adverse event.

**Extended Data Table 6:**
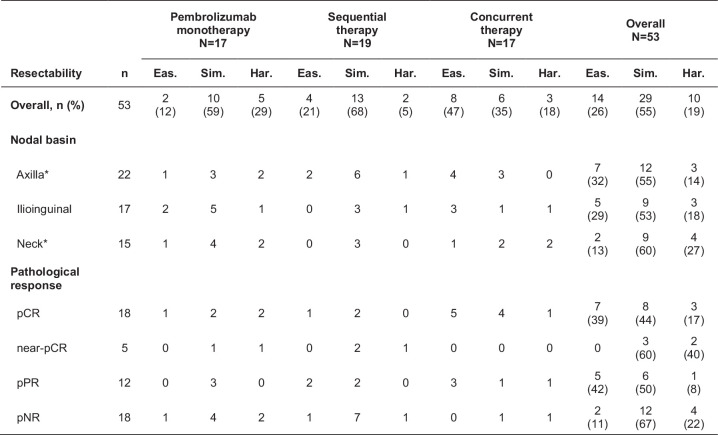
Ease of resection after neoadjuvant therapy

Surgeon’s assessment of whether week 6 surgery was easier than (Eas.), similar to (Sim.) or harder than (Har.) baseline assessment by nodal basin and pathological response. Fifty-three patients who underwent surgery and had completed resectability assessments at baseline and week 6 (post-operative) were included. *One patient had both axillary and neck nodal involvement. pCR, pathological complete response; pNR, pathological non-response; pPR, pathological partial response.

## Data Availability

De-identified data are available on reasonable request and after signing of a data transfer agreement with Melanoma Institute Australia. Requests for data sharing can be made to the corresponding author, G.V.L., including a research proposal that must be approved by the principal investigators of the three participating centers. The Background and Patient Information sections of the study protocol are provided in the Supplementary Information.
