# Supplementary material for: Neoadjuvant pembrolizumab, dabrafenib and trametinib in BRAFV600-mutant resectable melanoma: the randomized phase 2 NeoTrio trial
Source: Nat Med. 2024 Jun 21;30(9):2540–8. doi: 10.1038/s41591-024-03077-5 (PMC11405264; doi:10.1038/s41591-024-03077-5)
Supplement: Supplementary file 1 — Supplementary Table 1 and Supplementary Note (NeoTrio Protocol V4.0 sections). [file 41591_2024_3077_MOESM1_ESM.pdf]

nature medicine

Supplementary information https://doi.org/10.1038/s41591-024-03077-5

Neoadjuvant pembrolizumab, dabrafenib and trametinib in *BRAF*^{V600}-mutant resectable melanoma: the randomized phase 2 NeoTrio trial

In the format provided by the authors and unedited

Supplementary Data File

Supplementary Data **2**
Supplementary Data Table 1 | Definition of pathological response categories 2
NeoTrio Protocol V4.0 sections **3**
Title page 3
1 Background and Rationale 4
1.1 Introduction 4
1.2 Pharmaceutical and Therapeutic Background 4
1.3 Rationale for the Study 7
1.4 Conclusion 9
4 Patient Selection 11
4.1 Inclusion Criteria 11
4.2 Exclusion Criteria 13
11 References 15
Supplementary Data-only references **18**

Supplementary Data Table 1 | Definition of pathological response categories

	Category	Definition
MPR	pCR	No viable tumour cells in the tumour bed area
	near-pCR	>0 to ≤10% viable tumour cells in the tumour bed area
Non-MPR	pPR	>10 to ≤50% viable tumour cells in the tumour bed area
	pNR	>50% viable tumour cells in the tumour bed area

Definitions of pathological response categories per the International Neoadjuvant Melanoma Consortium.[1] MPR, major pathological response; pCR, pathological complete response; pNR, pathological non-response; pPR, pathological partial response.

Supplementary Data NeoTrio Protocol V4.0 Sections

A Phase II, Randomised, Open Label Study of Neoadjuvant Dabrafenib, Trametinib and / or Pembrolizumab in BRAF V600 Mutant Resectable Stage IIIB/C/D (excluding in-transit) Melanoma

Protocol Number	MIA2015/CT/179
Short Title	Neo Trio Study
Version Number	Version 4.0
Version Date	02 September 2021

Lead Investigator	Professor Georgina Long
Sponsor and Coordinating Centre	Melanoma Institute Australia
Address	Melanoma Institute Australia<br>The Poche Centre, 40 Rocklands Road<br>Wollstonecraft, NSW 2065<br>Australia

1 BACKGROUND AND RATIONALE

1.1 Introduction

The medical treatment options for advanced melanoma have accelerated in recent years, with resulting improvements in disease control and survival outcomes. The new therapeutic strategies come as a result of significant advances in the understanding of the immunomodulatory mechanisms and molecular biology of melanoma. The resulting new therapeutic strategies include oncogene-targeted therapy and immune checkpoint blockade, and these are now approved therapies that have transformed the routine clinical management for patients with metastatic melanoma. Both targeted and immunotherapy strategies have shown remarkable efficacy. However, most patients with advanced melanoma still die of their disease, and thus, there remains an urgent need to improve upon current therapies. Most patients with advanced disease eventually progress, and the question as to whether earlier treatment with systemic therapy after resection of all macroscopic melanoma (adjuvant therapy) improves long term survival is unknown. Furthermore, with the increased number of therapies utilised in melanoma, the question of optimal sequencing versus combination therapy remains unanswered. An efficient method of assessing drugs and combinations in humans is urgent and critical, particularly as combinations of molecularly targeted and/or immune therapies may have similar signals for efficacy in pre-clinical models, and recapitulation of the human immune system in animal models is limited.

Neoadjuvant clinical trials in patients with resectable but bulky stage III melanoma allows for the rapid evaluation of drug activity in humans utilising multiple clinical endpoints (FDG-PET, RECIST and pathological response; relapse-free survival; overall survival [OS]) and translational endpoints (multiple blood draws and melanoma tissue biopsies, along with complete resection of all melanoma after 6 weeks of therapy, which is analysed as outlined in Figure 1).

The planned dose of pembrolizumab for this study is 200 mg every 3 weeks (Q3W). Based on the totality of data generated in the Keytruda development program, 200 mg Q3W is the appropriate dose of pembrolizumab for adults across all indications and regardless of tumour type. As outlined below, this dose is justified by:

- Clinical data from 8 randomized studies in melanoma and NSCLC indications demonstrating flat dose- and exposure-efficacy relationships from 2 mg/kg Q3W to 10 mg/kg, representing an approximate 5- to 7.5-fold exposure range (refer to IB, Section 5.2.2),
- Population PK analysis showing that both fixed dosing and weight-based dosing provides similar control of PK variability with considerable overlap in the distributions of exposures, supporting suitability of 200 mg Q3W,
- Clinical data showing meaningful improvement in benefit-risk including overall survival at 200 mg Q3W across multiple indications, and
- Pharmacology data showing full target saturation in both systemic circulation (inferred from pharmacokinetic [PK] data) and tumour (inferred from physiologically-based PK [PBPK] analysis) at 200 mg Q3W.

1.2 Pharmaceutical and Therapeutic Background

1.2.1 Pembrolizumab

The importance of intact immune surveillance in controlling outgrowth of neoplastic transformation has been known for decades (Disis, 2010). Accumulating evidence shows a correlation between tumour-infiltrating lymphocytes (TILs) in cancer tissue and favourable prognosis in various malignancies(Hodi & Dranoff, 2010). In particular, the presence of CD8+ T-cells and the ratio of CD8+ effector T-cells / FoxP3+ regulatory T-cells seems to correlate with improved prognosis and long-term survival in many solid tumours including ovarian, colorectal, pancreatic, hepatocellular renal and melanoma.

The PD-1 receptor-ligand interaction is a major pathway hijacked by tumours to suppress immune control. The normal function of PD-1, expressed on the cell surface of activated T-cells under healthy conditions, is to down-modulate unwanted or excessive immune responses, including autoimmune reactions. PD-1 (encoded by the gene Pdcd1) is an Ig superfamily member related to CD28 and CTLA-4 which has been shown to negatively regulate antigen receptor signalling upon engagement of its ligands (PD-L1 and/or PD L2). The structure of murine PD-1 has been resolved. PD-1 and family members are type I transmembrane glycoproteins containing an Ig Variable-type (V-type) domain responsible for ligand binding and a cytoplasmic tail which is responsible for the binding of signalling molecules. The cytoplasmic tail of PD-1 contains 2 tyrosine-based signalling motifs, an immunoreceptor tyrosine-based inhibition motif (ITIM) and an immunoreceptor tyrosine-based switch motif (ITSM). Following T-cell stimulation, PD 1 recruits the tyrosine phosphatases SHP-1 and SHP-2 to the ITSM motif within its cytoplasmic tail, leading to the dephosphorylation of effector molecules such as CD3ζ, PKCθ and ZAP70 which are involved in the CD3 T-cell signalling cascade. The mechanism by which PD-1 down modulates T-cell responses is similar to, but distinct from that of CTLA-4 as both molecules regulate an overlapping set of signalling proteins. PD-1 was shown to be expressed on activated lymphocytes including peripheral CD4+ and CD8+ T-cells, B-cells, T regs and Natural Killer cells (Hodi & Dranoff, 2010). Expression has also been shown during thymic development on CD4-CD8- (double negative) T-cells as well as subsets of macrophages and dendritic cells. The ligands for PD-1 (PD-L1 and PD-L2) are constitutively expressed or can be induced in a variety of cell types, including non-haematopoietic tissues as well as in various tumours. Both ligands are type I transmembrane receptors containing both IgV- and IgC-like domains in the extracellular region and contain short cytoplasmic regions with no known signalling motifs. Binding of either PD-1 ligand to PD-1 inhibits T-cell activation triggered through the T-cell receptor. PD-L1 is expressed at low levels on various non-hematopoietic tissues, most notably on vascular endothelium, whereas PD-L2 protein is only detectably expressed on antigen-presenting cells found in lymphoid tissue or chronic inflammatory environments. PD-L2 is thought to control immune T-cell activation in lymphoid organs, whereas PD-L1 serves to dampen unwarranted T-cell function in peripheral tissues. Although healthy organs express little (if any) PD-L1, a variety of cancers were demonstrated to express abundant levels of this T-cell inhibitor. PD-1 has been suggested to regulate tumour-specific T-cell expansion in patients with melanoma (MEL) (Oble, Loewe, Yu, & Mihm, 2009). This suggests that the PD-1/PD-L1 pathway plays a critical role in tumour immune evasion and should be considered as an attractive target for therapeutic intervention.

Pembrolizumab is a potent and highly selective humanized monoclonal antibody (mAb) of the IgG4/kappa isotype designed to directly block the interaction between PD-1 and its ligands, PD-L1 and PD-L2. The US Food and Drug Administration (FDA) accelerated the approval of pembrolizumab for use for the treatment of patients with unresectable or metastatic melanoma, however only after progression on MAPK inhibitors (MAPKi) and/or ipilimumab. It is also approved by the therapeutic goods administration (TGA) and funded by the pharmaceutical benefits scheme (PBS) in Australia since 2015 for first-line treatment of patients with unresectable or metastatic melanoma, and as second-line for those with the BRAF mutation after progression on MAPK inhibitors (MAPKi).

In the largest melanoma phase 1 study of pembrolizumab, the response rate was 40% in those who were ipilimumab naïve and 28% in those who were ipilimumab refractory. The majority of responses were ongoing at data cut (18 April 2014), with a median duration of response not reached (range 8+-76+ weeks).(Ribas et al., 2014) Baseline tumour size was the only factor predictive of response and prognostic of survival in a multivariate analysis. Patients with increased PD-L1 expression were more likely to have an objective response compared to patients with no PD-L1 tumour expression, with response rates of 49% and 13%, respectively (P=0.0007). PD-L1 positivity was defined as staining in 1% or more of tumour cells.

While a small number of patients do gain long-lasting clinical benefit with immunotherapy, the overall survival advantage in all patients treated with these agents remains small and the potential toxicity is significant.

1.2.2 Dabrafenib and Trametinib

The mitogen-activated protein kinase (MAPK) pathway is a critical signal transduction pathway in normal and cancer cells. The MAPK pathway is a three -tiered kinase cascade consisting of the rapidly-activated fibrosarcoma kinase (RAF kinase), mitogen-activated extracellular signal-related kinase (MEK kinase), and extracellular signalling-regulated kinase (ERK or MAPK).

BRAF, one of three structurally related RAF-kinase isoforms (A-, B-, and C-RAF or RAF-1), is part of the MAPK-signal transduction pathway which controls cell cycle progression, differentiation, and survival. Under physiological conditions, signal transduction through the MAPK-pathway is tightly regulated through multiple negative feedback mechanisms. However, constitutive pathway activation through multiple genetic alterations is a hallmark of malignant tumours. For the serine-threonine kinase RAF alone over 45 cancer-associated mutations are currently known. Most of these mutations constitutively activate the RAF-kinase. In melanoma, more than 80% of the BRAF mutations cause a substitution of the amino acid glutamate (E) for valine (V) at position 600 (V600E) of the BRAF protein, whereas approximately 3-20% of melanoma mutations are a substitution of lysine (K) for valine at position 600 (V600K). The BRAF V600E mutation occurs at a high frequency in specific cancers, including approximately 60% of melanoma, 30 to 50% of papillary thyroid, 5 to 20% of colorectal, and approximately 30% of ovarian cancer.

Although BRAF-mutations were initially identified in premalignant, benign cutaneous nevi, there is overwhelming pre-clinical and clinical evidence that these mutations confer 'oncogenic addiction' to melanoma cells and are thus a key driver of advanced and metastatic disease and a prime target for therapeutic intervention with targeted small-molecule inhibitors.(Nissan & Solit, 2011) While the introduction of BRAF inhibitors represent a significant advance in the treatment of BRAF V600 mutation-positive metastatic melanoma patients (Chapman et al., 2011), limitations of this novel therapy have already been identified. As has been the pattern with other highly selective small molecule kinase inhibitors, the rapid onset of drug resistance restricts the efficacy of vemurafenib and limits the median duration of response to only 6.7 months (data from the vemurafenib Phase III study BRIM3). Understanding the specific mechanisms of resistance to BRAF-inhibitors is critical for the development of more effective strategies to inhibit the MAPK-pathway in order to delay or prevent the onset of resistance in BRAF-mutant melanoma.

In a majority of cell models and melanoma samples, acquired resistance to BRAF inhibitors was associated with a reactivation of the MAPK-pathway indicating that the 'addiction' to this pathway remains unchanged (Alcala & Flaherty, 2012). In these resistant BRAF mutant melanomas, the MAPK-pathway can be reactivated through secondary activating mutations of the upstream NRAS- or the downstream MEK1-kinase or an overexpression of the RAF1- and COTkinase. In addition, activation of further upstream RTKs most probably due to alterations in molecular feedback loops affecting in particular the IGF-IR (insulin-like-growth factor receptor) and the PDGFR (platelet-derived growth factor) have also been detected.

Although all of these molecular events enable the melanoma cell to circumvent BRAF-inhibition in order to re-activate the MAPK-pathway, this activation renders most of the BRAF-inhibitor resistant tumours susceptible to an inhibition of the downstream MEK-kinase. Experimental data generated with a BRAF- and MEK-inhibitor combination therapy in BRAF-mutant melanoma cell lines in vitro and xenografts in vivo support this concept by demonstrating activity of the combination therapy in models of acquired BRAF resistance. More importantly, superior anti-tumour activity of the BRAF- and MEK inhibitor combination as compared to each agent as monotherapy was also observed in BRAF-sensitive models.

These data clearly indicate that a concomitant and more potent inhibition of the MAPK-pathway at the critical level of the BRAF- and MEK-kinases leads to a more pronounced tumour inhibition, thus significantly delaying the onset of resistance. In addition, pre -clinical safety data obtained with this combination therapy in a rat-model indicate that the potential for proliferative skin lesions and secondary cutaneous malignancies is reduced in comparison to treatment with a BRAF-inhibitor alone (Su et al., 2012).

Dabrafenib, a 4-(3-aminosulfonylphenyl)-5-(pyrimidin-3-yl) thiazole, is a potent and selective inhibitor of B-RAF kinase activity with a mode of action consistent with adenosine triphosphate (ATP)-competitive inhibition and is approved as monotherapy in BRAF V600E-mutant advanced/metastatic melanoma. Trametinib, a pyrido - pyrimidine derivative, is a potent and highly selective allosteric non-competitive inhibitor of MEK1/MEK2 activation and kinase activity has been approved as monotherapy in BRAF (V600E)-mutant and BRAF (V600K)-mutant melanoma. The safety, tolerability, PK and clinical activity of trametinib + dabrafenib combination therapy has been evaluated in patients with BRAF-mutant melanoma in a Phase I/II study (Flaherty et al., 2012), and demonstrated good efficacy with 76% ORR and 9.4 month median PFS at the highest dose combination.

The use of combined BRAF and MEK inhibition is now standard care for patients with V600 BRAF mutant melanoma. The BRAF inhibitor dabrafenib combined with the MEK inhibitor trametinib were approved for use in unresectable stage IIIC and stage IV melanoma by the Food and Drug Administration (FDA) in the USA in 2014. In Australia, the combination is approved by the TGA in 2014 and funded by the pharmaceutical benefits scheme (PBS). Three phase 3 studies have demonstrated superiority of the combination over single agent BRAF inhibition for response, progression free survival (PFS) and overall survival (OS). (Larkin et al., 2014; Georgina V. Long et al., 2015; Georgina V. Long et al., 2014; G. V. Long et al., 2016; Caroline Robert et al., 2015; C. Robert et al., 2015; C. Robert et al., 2014) Response rates for the combination range from 64-68% and the median PFS is 9-12 months. In the phase 2 study of dabrafenib combined with trametinib in metastatic melanoma, the median OS was 25.1 months in BRAF inhibitor–naive patients and approximately 20% were progression free at 3 years. Durable responses occurred in patients with good prognostic features at baseline, which may be predictive.(Georgina V. Long et al., 2015; G. V. Long et al., 2016) The development of resistance to BRAF inhibitors, even in combination with a MEK inhibitor, remains a challenge (Johnson et al., 2015; Rizos et al., 2014; Shi et al., 2014).

The dabrafenib and trametinib combination therapy is currently being studied in the neoadjuvant and adjuvant setting in patients with bulky Stage IIIB/C melanoma. Early results (Saw et al., 2016) prior to the completion of follow up suggests neoadjuvant combined targeted therapy in these patients results in promising reduction in tumour burden prior to surgery. The outcome of adjuvant treatment should be known in 2018.

1.3 Rationale for the Study

Surgery remains the standard of care for resectable Stage III melanoma, despite the recent drug therapy advances described above. The Food and Drug Administration (FDA) has recently expanded the approved use of Yervoy™ (ipilimumab) to include a new use as adjuvant therapy for patients with stage III melanoma, to lower the risk of relapse following surgery. Neoadjuvant therapy in this group of patients may also result in improved survival rates and in the duration of local and distant disease control, with reduced surgical morbidity and the potential for early elimination of microscopic metastatic disease.

There is an emerging and rapidly growing evidence base of the value of combining targeted and immunotherapies in a number of histological subtypes of cancers. The support for a potential synergy between the two treatment modalities has been established (Cooper et al., 2014; Dennie T. Frederick et al., 2013; Wilmott et al., 2012) as has the increased toxicity profile (L. Robert, Ribas, & Hu-Lieskovan, 2016). Both single agent BRAF inhibitors and combined BRAF and MEK inhibitors induce a marked clonal T cell infiltrate in responding melanoma metastases early during treatment (day 7-15), which is transient, and is not present at progression. Concurrently, melanoma tumour antigen and PDL1 expression increase early during treatment. (D. T. Frederick et al., 2013)

Clinical trials of combined modalities are underway in melanoma, the majority in the metastatic setting. Of critical importance to this study is the 3-part Phase I/II trial of pembrolizumab, dabrafenib and trametinib in metastatic melanoma (MK-3475-022/KEYNOTE 022, NCT02130466) in which the same drug combination will be administered. Recruitment to Part 3 is underway in this randomised trial of

pembrolizumab, dabrafenib and trametinib versus saline placebo with dabrafenib and trametinib, in approximately 120 patients worldwide. Parts 1 and 2 of KEYNOTE 022 evaluated the safety, dosing and preliminary efficacy of pembrolizumab in combination with dabrafenib and trametinib in BRAF mutant melanoma and pembrolizumab with trametinib in BRAF mutation negative melanoma. The same dose of the triple combination in BRAF mutant melanoma will be used in this current trial in the neoadjuvant setting: for one week duration followed by pembrolizumab or for 6 weeks concurrently with pembrolizumab

Early phase studies testing combined targeted therapies or combined immunotherapies are also underway in the neoadjuvant setting. However, this study will be one of the early trials to test combined drug classes (based on search of NIH Clinical Trials Registry April 2017). Refer to Table 1 for further details.

Table 1 Current Clinical Trials of Systemic Combination Therapies and Drug Classes for Melanoma in the Neoadjuvant Setting as of April 2017 (displayed in sample size order)

Sample Size	Drug Combinations	Status	Clinicaltrials.gov ID
n=20	Vemurafenib + Cobimetinib	R	NCT03005639
n=20	Vemurafenib + Cobimetinib	R	NCT02036086
n=20	Ipilimumab + Nivolumab neoadjuvant **or** adjuvant	A, NR	NCT02437279
n=30	Dabrafenib **or** Dabrafenib + Trametinib	R	NCT01978236
n=30	Pembrolizumab + High Dose IFN-alfa2b	R	NCT02339324
n=30	Ipilimumab 10mg/kg alone **or** Ipilimumab 3mg/kg + high-dose IFN-α-2	A, NR	NCT01608594
n=35	Dabrafenib + Trametinib	A, NR	NCT01972347
n=40	Nivolumab alone **or** Nivolumab + Ipilimumab	R	NCT02519322
n=66	Ipilimumab + Nivolumab **or** Nivolumab Alone	R	NCT02736123
n=78	Dabrafenib + Trametinib	R	NCT02231775
n=90	Ipilimumab + Nivolumab in 3 dose combinations	R	NCT02977052
n=110	Vemurafenib + Cobimetinib	R	NCT02303951

Key: **R**=currently recruiting patients; **A, NR**=Active, no longer recruiting patients (recruitment complete)

It is unknown whether there is potential for converting a subset of patients who fail either immunotherapy or targeted therapy alone into long-term responders by treating with PD-1 inhibitors in conjunction with MAPK targeted therapies. Furthermore, it is unclear whether the PD-1 inhibitor would be best combined sequentially or concurrently with MAPK inhibitors (Ackerman et al., 2014). Mouse models have provided a clear rational for combining these treatments upfront, however there is no human tissue evidence to guide best combination strategies. (Cooper, Frederick, Ahmed, & Wargo, 2013; Cooper et al., 2014)

The question of how best to maximize clinical outcome via concurrent versus sequential targeted and immune therapy may be explored efficiently in the human neoadjuvant setting, with detailed interrogation of multiple biopsies early during treatment. Immunological, proteomic and genetic features in tissue and blood provide an in vivo assessment of tumour responsiveness to therapy. This may enable more selective application of therapeutic agents to patients who are more likely to benefit. Such findings would improve the therapeutic index and cost effectiveness of these agents. Earlier systemic therapy prior to surgery also means earlier targeting of distant micrometastases that could become the source of future disease relapse.

The rationale for this study design is therefore based on the hypothesis that one week of targeted therapy may be sufficient to induce an enhanced tumoral immunity to result in a higher pathological and RECIST response when followed sequentially with pembrolizumab, or when given in combination with pembrolizumab.

A current study of neoadjuvant dabrafenib and trametinib in the same patient population (NCT01972347), at the same institution and with a similar design, completed recruitment in early 2017. The last patient will have reached the end of the 52 week treatment phase in April 2018 in that study. It is anticipated that the data from that study will be of interest when assessing the clinical, pathological, surgical and metabolic outcomes in this current study. As will the adverse event profile and the biomarker analyses. Study NCT01972347 therefore provides a virtual 4th arm to this protocol – that of combined targeted therapy with no immunotherapy inclusion.

The potential for toxicities that could affect adherence to the combined study treatments are recognised, as additive, overlapping or unforeseen adverse events may occur with the triple combination. The adverse event profiles and safety-related interruption to treatment will therefore be assessed in conjunction with the objective responses.

1.4 Conclusion

The clinical and translational findings from this study have the potential to inform rational decisions regarding combinations of treatment both in the metastatic and the adjuvant settings. This is a critical study to inform future practice and future phase 3 clinical trials. The translational research performed on human tissue biopsies and blood samples will provide mechanistic information to guide the selection of optimal combinations of therapies for phase 3 studies in the advanced and the adjuvant setting.

Figure 1 Overview of the three study treatment arms and the key assessment timelines

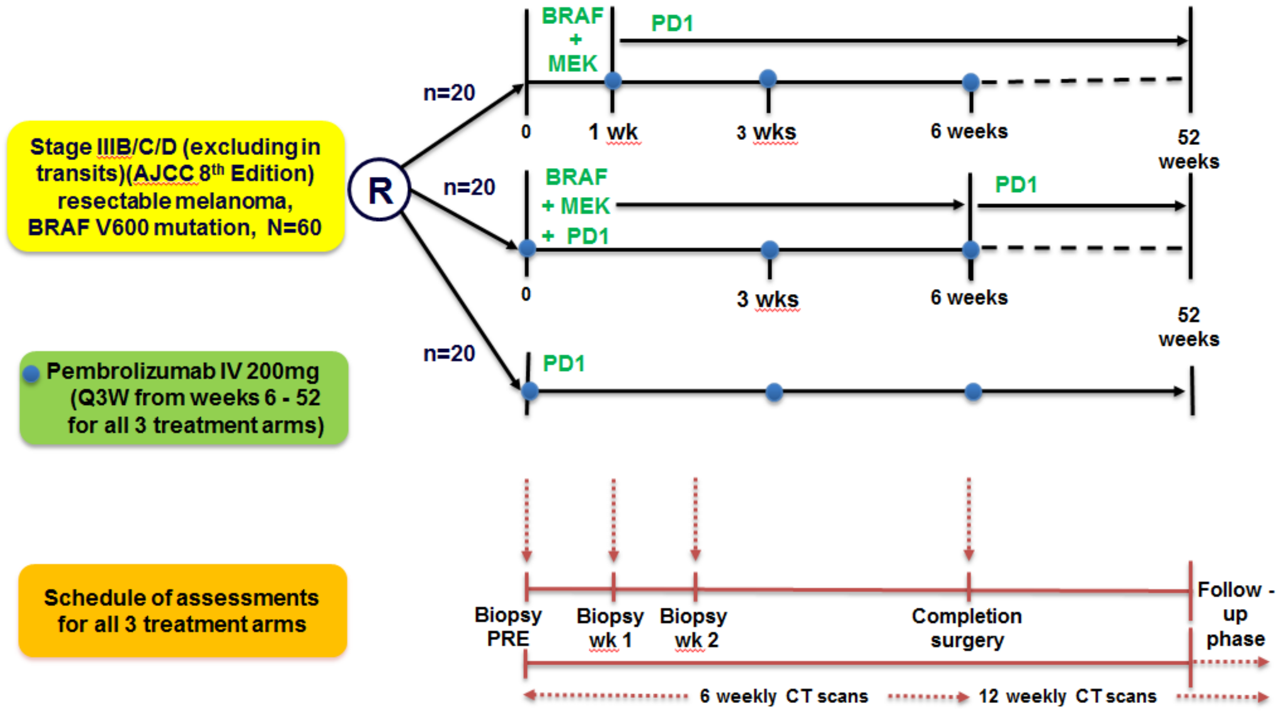

10

4 PATIENT SELECTION

A total of 60 patients will be randomised to receive one of three treatment regimes, with 20 patients in each treatment arm. Patients who drop out of any arm before the completion of one dose of study drug due to withdrawn consent, non-compliance with protocol, a new concurrent illness that renders continuation unsafe or those who are lost to follow up will be replaced. Patients who have to discontinue study treatment before 6 weeks because of drug related toxicities, disease progression or death will not be replaced.

Patients who meet all the inclusion criteria and have none of the exclusion criteria as listed below will be randomised.

4.1 Inclusion Criteria

1. ≥18 years of age.
2. Written informed consent.
3. Histologically confirmed, resectable AJCC (8th edition) stage IIIB or IIIC or IIID (refer to Appendix E) for full staging criteria (Gershenwald et al., 2017).At baseline, patients may have a primary melanoma in addition to nodal disease. At baseline, there must be sufficient cutaneous or nodal disease to enable multiple excisional or core biopsies (at baseline, day 8, and day 15).

   'Resectable' tumours are defined as having no significant vascular, central nervous system or bony involvement. Only cases where a complete surgical resection with tumour-free margins can safely be achieved are defined as 'resectable'. Patients who may not have sufficient disease to enable multiple biopsies at days 8 and 15 will not be excluded, however the intention of the study is that at least one biopsy at these time points is required.
4. Measurable disease according to RECIST version 1.1 criteria (≥ 10mm longest diameter for primary [if applicable] lesions and ≥ 15mm in shortest diameter for lymph nodes) within 2 weeks of randomisation. CT is preferred for all lesions where possible. MRI brain and total body FDG PET imaging will be performed within this timeframe, but these will not be used for the primary purpose of measuring RECIST response.
5. BRAF V600 mutation positive on immunohistochemistry or a local molecular test (e.g. Oncofocus)
   a. A positive V600E immunohistochemistry stain at study entry should be formally quantified with a local molecular test following study entry (e.g. Oncofocus)
   b. Molecular BRAF mutation status should preferentially be confirmed using tissue taken from the presenting stage III disease. Alternatively, archival primary tissue is also acceptable to confirm BRAF mutation status
6. Able to swallow and retain oral medication
7. Eastern Cooperative Oncology Group (ECOG) performance status of 0-1
8. Demonstrated adequate organ function as defined in the table below:

System	Laboratory Value
**Haematological**	
• Absolute neutrophil count (ANC)	≥1.5 10^9/L
• Platelets	≥100 10^9/L
• Haemoglobin	≥90g/L

System	Laboratory Value
Renal	
• Serum creatinine <u>OR</u>	≤1.5 X upper limit of normal (ULN) <u>OR</u>
• Measured or calculated creatinine clearance ‡	≥60 mL/min for patient with creatinine levels > 1.5 X institutional upper limit of normal (ULN)
• (GFR can also be used in place of creatinine or CrCl)	
Hepatic	
• Serum total bilirubin	≤ 1.5 X ULN <u>OR</u><br>Direct bilirubin ≤ ULN for patients with total bilirubin levels > 1.5 ULN
• AST (SGOT) and ALT (SGPT)	≤ 2.5 X ULN <u>OR</u><br>≤ 5 X ULN for patients with liver metastases
• Albumin	≥25 g/L
Coagulation	
• International Normalized Ratio (INR) or Prothrombin Time (PT)	≤1.5 X ULN unless patient is receiving anticoagulant therapy as long as PT or PTT is within therapeutic range of intended use of anticoagulants
• Activated Partial Thromboplastin Time (aPTT)	≤1.5 X ULN unless patient is receiving anticoagulant therapy as long as PT or PTT is within therapeutic range of intended use of anticoagulants
‡ Creatinine clearance should be calculated per institutional standard.	

9. Anticipated life expectancy of > 12 months.
10. Women of childbearing potential: a negative serum pregnancy test within 72 hours of first dose of study treatment and effective contraception from 14 days prior to study treatment until 4 months after the last dose. 'Effective' contraception shall mean:
    a. intrauterine device with a documented failure rate of less than 1% per year.
    b. vasectomised partner who is sterile prior to the female partner patient's commencement of study treatment and is the sole sexual partner for that female.
    c. double barrier contraception: male condom and occlusive cap (diaphragm or cervical /vault caps).

    Women not of 'childbearing potential' are defined as any female who has had a documented hysterectomy, bilateral oopherectomy or bilateral tubal ligation or any female who is post-menopausal (≥ one year without menses and >50 years of age in the absence of hormone replacement therapy).

    Hormonal contraception alone is not recommended for the prevention of pregnancy as concomitant treatment with dabrafenib reduces the efficacy of oral contraceptives.

    Refer to Section 5.16.2 for further information on avoiding pregnancy.
11. Men with a female partner of childbearing potential to use effective contraception from 14 days prior to study treatment until 4 months after the last dose. 'Effective' contraception shall mean:
    a. Documented vasectomy and sterility
    b. In the partner - intrauterine device with a documented failure rate of less than 1% per year

c. Double barrier contraception: male condom and occlusive cap (diaphragm or cervical/vault caps).

Refer to Section 5.16.2 for further information on avoiding pregnancy.

4.2 Exclusion Criteria

1. Uveal or mucosal melanoma.
2. Prior anti-cancer treatment for melanoma, **except** for the following:
    a. Surgery for a primary melanoma or previous stage III melanoma,
    b. Adjuvant radiotherapy to the primary melanoma resected site or adjuvant radiotherapy to lymph nodes for previous Stage III disease, previous adjuvant interferon or ipilimumab for resected stage II or III melanoma,
    c. Previous adjuvant treatment with PD1 inhibitors or BRAF/MEK inhibitors is not permitted.
3. Received any investigational drug within 28 days or 5 half-lives of the planned first dose of this study treatment.
4. Known immediate or delayed hypersensitivity reaction or idiosyncrasy to drugs chemically related to the study treatments, their excipients and / or dimethyl sulfoxide (DMSO).
5. Active infection requiring systemic therapy.
6. Current use of any prohibited medication as described in Section 5.15.2.
7. Active autoimmune disease or a documented history of autoimmune disease or a syndrome requiring systemic steroids or immunosuppressive agents. Patients with the following are permitted to enrol:
    a. vitiligo,
    b. type I diabetes mellitus,
    c. residual hypothyroidism due to an autoimmune condition only requiring, and stable on hormone replacement,
    d. resolved childhood asthma or atopy,
    e. psoriasis not requiring systemic treatment,
    f. or autoimmune conditions not expected to recur in the absence of an external trigger.
8. A requirement for chronic systemic steroid therapy (> 10mg/kg per day of prednisone or equivalent) within two weeks before the planned first dose of study treatment or any on any other form of immunosuppressive treatment. Patients who require inhaled or intranasal corticosteroids (with minimal systemic absorption) may be continued if the patient is on a stable dose. Non-absorbed intra-articular steroid injections will also be permitted.
9. A known history of another malignancy or concurrent malignancy unless the patient is disease-free for a minimum of 1 year, is completely treated and is at low-risk of recurrence. The time requirement does not apply for patients with successful definitive resection or curative treatment of:
    a. Non-melanoma skin cancer (e.g. basal cell or squamous cell carcinoma of the skin),
    b. superficial bladder cancer,
    c. in situ carcinoma of the cervix,
    d. in situ breast cancer,
    e. atypical melanocytic hyperplasia or melanoma in situ
    f. other in situ carcinomas,
    g. multiple primary melanomas, or other treated low risk tumours.

10. Known to be HIV, hepatitis B or C virus positive status or history of active tuberculosis (testing prior to randomisation is not required).

11. Administration of a live vaccine or live-attenuated vaccine with 30 days of planned first dose of study treatment. Seasonal influenza vaccines for injection are generally inactivated flu vaccines and are allowed, however intranasal influenza vaccines (e.g., Fluad®) are live attenuated vaccines, and are not allowed. Any vaccine is cautionary within 30 days of starting study treatment. Administration of killed vaccines is allowed.

12. Patients with a history or evidence of cardiovascular risk including any of the following:
    a. QT interval corrected for heart rate using the Bazett formula ≥480 msec, a diagnosis of long QT syndrome (Roman-Ward or Jervell Lange-Nielsen syndromes),
    b. Taking medications known to prolong the QT interval,
    c. Uncorrectable electrolyte abnormal abnormality (e.g. hypo- or hyperkalaemia, hypomagnesaemia, hypocalcaemia),
    d. Uncontrolled arrhythmias, with the exception of atrial fibrillation which is controlled for > 30 days prior to randomisation,
    e. Patients with implanted cardioverter/defibrillators,
    f. Acute coronary syndromes (including myocardial infarction or unstable angina), coronary angioplasty or stenting within 6 months prior to randomisation,
    g. A history or current evidence of NYHA ≥Grade 2 congestive heart failure,
    h. A current left ventricular ejection fraction (LVEF) below than the lower limit of normal (LLN).
    i. Any abnormal cardiac valve morphology documented by echocardiogram which in the opinion of the investigator could interfere with the patient's safety,
    j. Treatment-refractory hypertension defined as a systolic blood pressure of >140 mm Hg and/or a diastolic pressure of >90 mm Hg, which cannot be controlled by anti-hypertensive treatment.

13. Evidence or a risk of retinal vein occlusion or central serous retinopathy, including:
    a. Presence of predisposing factors to RVO or CSR (e.g., uncontrolled glaucoma or ocular hypertension, uncontrolled hypertension, uncontrolled diabetes mellitus, or a history of hyperviscosity or hypercoagulability syndromes),
    b. Visible retinal pathology as assessed by ophthalmic examination that is considered a risk factor for RVO or CSR, such as evidence of new optic disc cupping,
    c. Intraocular pressure > 21 mm Hg as measured by tonography,
    d. Evidence of new visual field defects on automated perimetry.

14. History or evidence of interstitial lung disease or active non-infectious pneumonitis.

15. Serious or unstable pre-existing medical conditions or other conditions that could interfere with the patient's safety, consent, or compliance.

16. Has known psychiatric or substance abuse disorders that would interfere with cooperation with the requirements of the trial.

17. Has received prior therapy with an anti-PD-1, anti-PD-L1, or anti-PD-L2 agent or an agent directed to another co-inhibitory T-cell receptor (i.e. OX-40). Anti CTLA-4 given in the adjuvant setting is permitted.

18. Pregnant or breastfeeding females, or expecting to conceive or father children within the projected period of study treatment (52 weeks followed by 4 months following end of study treatment).

19. History of In-transit metastases within the last 6 months

11 REFERENCES

A'Hern, R. (2001). Sample size tables for exact single-stage phase II designs. *Statistics in Medicine, 20*, 859-866.

Ackerman, A., Klein, O., McDermott, D. F., Wang, W., Ibrahim, N., Lawrence, D. P., . . . Sullivan, R. J. (2014). Outcomes of patients with metastatic melanoma treated with immunotherapy prior to or after BRAF inhibitors. *Cancer, 120*(11), 1695-1701. doi:10.1002/cncr.28620

Alcala, A. M., & Flaherty, K. T. (2012). BRAF inhibitors for the treatment of metastatic melanoma: clinical trials and mechanisms of resistance. *Clin Cancer Res, 18*(1), 33-39. doi:10.1158/1078-0432.ccr-11-0997

Chapman, P. B., Hauschild, A., Robert, C., Haanen, J. B., Ascierto, P., Larkin, J., . . . McArthur, G. A. (2011). Improved Survival with Vemurafenib in Melanoma with BRAF V600E Mutation. *New England Journal of Medicine, 364*(26), 2507-2516. doi:doi:10.1056/NEJMoa1103782

Cooper, Z. A., Frederick, D. T., Ahmed, Z., & Wargo, J. A. (2013). Combining checkpoint inhibitors and BRAF-targeted agents against metastatic melanoma. *Oncoimmunology, 2*(5).

Cooper, Z. A., Juneja, V. R., Sage, P. T., Frederick, D. T., Piris, A., Mitra, D., . . . Wargo, J. A. (2014). Response to BRAF inhibition in melanoma is enhanced when combined with immune checkpoint blockade. *Cancer Immunol Res, 2*(7), 643-654. doi:10.1158/2326-6066.cir-13-0215

Disis, M. L. (2010). Immune regulation of cancer. *J Clin Oncol, 28*(29), 4531-4538. doi:10.1200/jco.2009.27.2146

Eisenhauer, E. A., Therasse, P., Boggaerts, J., Schwartz, L. H., Sargent, D., Ford, R., . . . Verweij, J. (2009). New response evaluation criteria in solid tumours: Revised RECIST guideline (version 1.1). *Eur J Cancer, 45*, 228-247.

Flaherty, K. T., Robert, C., Hersey, P., Nathan, P., Garbe, C., Milhem, M., . . . Schadendorf, D. (2012). Improved Survival with MEK Inhibition in BRAF-Mutated Melanoma. *New England Journal of Medicine, 367*(2), 107-114. doi:doi:10.1056/NEJMoa1203421

Frederick, D. T., Piris, A., Cogdill, A. P., Cooper, Z. A., Lezcano, C., Ferrone, C. R., . . . Wargo, J. A. (2013). BRAF Inhibition Is Associated with Enhanced Melanoma Antigen Expression and a More Favorable Tumor Microenvironment in Patients with Metastatic Melanoma. *Clinical Cancer Research*. doi:10.1158/1078-0432.CCR-12-1630

Frederick, D. T., Piris, A., Cogdill, A. P., Cooper, Z. A., Lezcano, C., Ferrone, C. R., . . . Wargo, J. A. (2013). BRAF Inhibition Is Associated with Enhanced Melanoma Antigen Expression and a More Favorable Tumor Microenvironment in Patients with Metastatic Melanoma. *Clinical Cancer Research*. doi:10.1158/1078-0432.ccr-12-1630

Gershenwald, J. E., Scolyer, R. A., Hess, K. R., Thompson, J. F., Long, G. V., Ross, M. I., . . . Sondak, V. K. (2017). Melanoma of the Skin. In *AJCC Cancer Staging Manual* (8th ed., pp. 563-585): American Joint Committee on Cancer.

Hodi, F. S., & Dranoff, G. (2010). The biologic importance of tumor-infiltrating lymphocytes. *J Cutan Pathol, 37 Suppl 1*, 48-53. doi:10.1111/j.1600-0560.2010.01506.x

Hodi, F. S., Hwu, W. J., Kefford, R., Weber, J. S., Daud, A., Hamid, O., . . . Wolchok, J. D. (2016). Evaluation of Immune-Related Response Criteria and RECIST v1.1 in Patients With Advanced Melanoma Treated With Pembrolizumab. *J Clin Oncol, 34*(13), 1510-1517. doi:10.1200/jco.2015.64.0391

International Conference on Harmonisation of Technical Requirements for Registration of Pharmaceuticals for Human Use. (2000). *Note for Guidance on Clinical Safety Data Management: Definitions and Standards for Expedited Reporting (CPMP/ICH/377/95) Annotated with TGA Comments*. TGA Publications Office.

Johnson, D. B., Menzies, A. M., Zimmer, L., Eroglu, Z., Ye, F., Zhao, S., . . . Schadendorf, D. (2015). Acquired BRAF inhibitor resistance: A multicenter meta-analysis of the spectrum and frequencies, clinical behaviour, and phenotypic associations of resistance mechanisms. *Eur J Cancer, 51*(18), 2792-2799. doi:10.1016/j.ejca.2015.08.022

Larkin, J., Ascierto, P. A., Dréno, B., Atkinson, V., Liszkay, G., Maio, M., . . . Ribas, A. (2014). Combined Vemurafenib and Cobimetinib in BRAF-Mutated Melanoma. *New England Journal of Medicine, 371*(20), 1867-1876. doi:doi:10.1056/NEJMoa1408868

Long, G. V., Stroyakovskiy, D., Gogas, H., Levchenko, E., de Braud, F., Larkin, J., . . . Flaherty, K. (2015). Dabrafenib and trametinib versus dabrafenib and placebo for Val600 BRAF-mutant melanoma: a multicentre, double-blind, phase 3 randomised controlled trial. *The Lancet, 386*(9992), 444-451. doi:10.1016/S0140-6736(15)60898-4

Long, G. V., Stroyakovskiy, D., Gogas, H., Levchenko, E., de Braud, F., Larkin, J., . . . Flaherty, K. (2014). Combined BRAF and MEK Inhibition versus BRAF Inhibition Alone in Melanoma. *New England Journal of Medicine, 371*(20), 1877-1888. doi:doi:10.1056/NEJMoa1406037

Long, G. V., Weber, J. S., Infante, J. R., Kim, K. B., Daud, A., Gonzalez, R., . . . Flaherty, K. T. (2016). Overall Survival and Durable Responses in Patients With BRAF V600-Mutant Metastatic Melanoma Receiving Dabrafenib Combined With Trametinib. *J Clin Oncol*. doi:10.1200/jco.2015.62.9345

National Health and Medical Research Council. (2016). *Safety monitoring and reporting in clinical trials involving therapeutic goods*. Canberra:: National Health and Medical Research Council.

Nissan, M. H., & Solit, D. B. (2011). The "SWOT" of BRAF inhibition in melanoma: RAF inhibitors, MEK inhibitors or both? *Curr Oncol Rep, 13*(6), 479-487. doi:10.1007/s11912-011-0198-4

Oble, D. A., Loewe, R., Yu, P., & Mihm, M. C., Jr. (2009). Focus on TILs: prognostic significance of tumor infiltrating lymphocytes in human melanoma. *Cancer Immun, 9*, 3.

Ribas, A. F., Hodi, S., Kefford, R., Hamid, O., Daud, A., Wolchok, J. D., . . . Robert, C. (2014). Efficacy and safety of the anti-PD-1 monoclonal antibody MK-3475 in 411 patients (pts) with melanoma (MEL). *Journal of Clinical Oncology, 32*(5s), Abstr 9003.

Rizos, H., Menzies, A. M., Pupo, G. M., Carlino, M. S., Fung, C., Hyman, J., . . . Long, G. V. (2014). BRAF inhibitor resistance mechanisms in metastatic melanoma: spectrum and clinical impact. *Clin Cancer Res, 20*(7), 1965-1977.

Robert, C., Karaszewska, B., Schachter, J., Rutkowski, P., Mackiewicz, A., Stroiakovski, D., . . . Schadendorf, D. (2015). Improved Overall Survival in Melanoma with Combined Dabrafenib and Trametinib. *New England Journal of Medicine, 372*(1), 30-39. doi:doi:10.1056/NEJMoa1412690

Robert, C., Karaszewska, B., Schachter, J., Rutkowski, P., Mackiewicz, A., Stroiakovski, D., . . . Schadendorf, D. (2015). Improved overall survival in melanoma with combined dabrafenib and trametinib. *N Engl J Med, 372*(1), 30-39. doi:10.1056/NEJMoa1412690

Robert, C., Karaszewska, B., Schachter, J., Rutkowski, P., Mackiewicz, A., Stroyakovskiy, D., . . . Schadendorf, D. (2014). COMBI-v: A randomised, open-label, Phase III study comparing the combination of dabrafenib (D) and trametinib (T) to vemurafenib (V) as first-line therapy in patients (pts) with unresectable or metastatic BRAF V600E/K mutation-positive cutaneous melanoma. *Annals of Oncology 25*(5), 1-41.

Robert, L., Ribas, A., & Hu-Lieskovan, S. (2016). Combining targeted therapy with immunotherapy. Can 1+1 equal more than 2? *Semin Immunol*. doi:10.1016/j.smim.2016.01.001

Saw, R., Menzies, A., Guminski, A., Nieweg., O., Shannon, K., Gonzalez, M., . . . Long, G. (2016). Phase 2 study of neoadjuvant dabrafenib + trametinib (D+T) for resectable stage IIIb/c BRAF-V600 mutation positive melanoma. *J Clin Oncol, 34*, suppl; abstr 9583.

Shi, H., Hugo, W., Kong, X., Hong, A., Koya, R. C., Moriceau, G., . . . Lo, R. S. (2014). Acquired resistance and clonal evolution in melanoma during BRAF inhibitor therapy. *Cancer Discov, 4*(1), 80-93.

Su, F., Viros, A., Milagre, C., Trunzer, K., Bollag, G., Spleiss, O., . . . Marais, R. (2012). RAS mutations in cutaneous squamous-cell carcinomas in patients treated with BRAF inhibitors. *N Engl J Med, 366*(3), 207-215. doi:10.1056/NEJMoa1105358

Wilmott, J. S., Long, G. V., Howle, J. R., Haydu, L. E., Sharma, R. N., Thompson, J. F., . . . Scolyer, R. A. (2012). Selective BRAF inhibitors induce marked T-cell infiltration into human metastatic melanoma. *Clinical Cancer Research, 18*(5), 1386-1394. doi:10.1158/1078-0432.CCR-11-2479

Wolchok, J. D., Hoos, A., O'Day, S., Weber, J. S., Hamid, O., Lebbé, C., . . . Hodi, F. S. (2009). Guidelines for the Evaluation of Immune Therapy Activity in Solid Tumors: Immune-Related Response Criteria. *Clinical Cancer Research, 15*(23), 7412-7420. doi:10.1158/1078-0432.ccr-09-1624

Supplementary Data-only references

1 Tetzlaff, M. T. *et al.* Pathological assessment of resection specimens after neoadjuvant therapy for metastatic melanoma. *Ann Oncol* **29**, 1861–1868 (2018). https://doi.org:10.1093/annonc/mdy226
