# Supplementary material for: Neoadjuvant pembrolizumab, dabrafenib and trametinib in BRAFV600-mutant resectable melanoma: the randomized phase 2 NeoTrio trial
Source: Nat Med. 2024 Jun 21;30(9):2540–8. doi: 10.1038/s41591-024-03077-5 (PMC11405264; doi:10.1038/s41591-024-03077-5)
Supplement: Supplementary file 2 — Reporting Summary [file 41591_2024_3077_MOESM2_ESM.pdf]

nature portfolio

Corresponding author(s): Georgina V Long

Last updated by author(s): May 10, 2024

Reporting Summary

Nature Portfolio wishes to improve the reproducibility of the work that we publish. This form provides structure for consistency and transparency in reporting. For further information on Nature Portfolio policies, see our Editorial Policies and the Editorial Policy Checklist.

Statistics

For all statistical analyses, confirm that the following items are present in the figure legend, table legend, main text, or Methods section.

n/a	Confirmed	
☐	☒	The exact sample size (*n*) for each experimental group/condition, given as a discrete number and unit of measurement
☒	☐	A statement on whether measurements were taken from distinct samples or whether the same sample was measured repeatedly
☐	☒	The statistical test(s) used AND whether they are one- or two-sided<br>*Only common tests should be described solely by name; describe more complex techniques in the Methods section.*
☐	☒	A description of all covariates tested
☐	☒	A description of any assumptions or corrections, such as tests of normality and adjustment for multiple comparisons
☐	☒	A full description of the statistical parameters including central tendency (e.g. means) or other basic estimates (e.g. regression coefficient) AND variation (e.g. standard deviation) or associated estimates of uncertainty (e.g. confidence intervals)
☐	☒	For null hypothesis testing, the test statistic (e.g. *F*, *t*, *r*) with confidence intervals, effect sizes, degrees of freedom and *P* value noted<br>*Give P values as exact values whenever suitable.*
☒	☐	For Bayesian analysis, information on the choice of priors and Markov chain Monte Carlo settings
☒	☐	For hierarchical and complex designs, identification of the appropriate level for tests and full reporting of outcomes
☒	☐	Estimates of effect sizes (e.g. Cohen's *d*, Pearson's *r*), indicating how they were calculated

Our web collection on statistics for biologists contains articles on many of the points above.

Software and code

Policy information about availability of computer code

Data collection	Data collection was performed by the Clinical Trials unit at Melanoma Institute Australia and participating sites.
Data analysis	All data and statistical analyses were performed using SAS (version 9.4) and R (version 4.1.3) by authors SNL and JT.

For manuscripts utilizing custom algorithms or software that are central to the research but not yet described in published literature, software must be made available to editors and reviewers. We strongly encourage code deposition in a community repository (e.g. GitHub). See the Nature Portfolio guidelines for submitting code & software for further information.

Data

Policy information about availability of data

All manuscripts must include a data availability statement. This statement should provide the following information, where applicable:

- Accession codes, unique identifiers, or web links for publicly available datasets
- A description of any restrictions on data availability
- For clinical datasets or third party data, please ensure that the statement adheres to our policy

De-identified data are available on reasonable request, and after signing of a data transfer agreement with Melanoma Institute Australia. Requests for data sharing can be made to the corresponding author, Georgina V Long, including a research proposal that must be approved by the principal investigators of the three participating centres. The Background and Patient Information sections of the study protocol are provided in the Supplementary Information.

Research involving human participants, their data, or biological material

Policy information about studies with human participants or human data. See also policy information about sex, gender (identity/presentation), and sexual orientation and race, ethnicity and racism.

Reporting on sex and gender	We report self-reported biological sex in the study, and did not collect or report gender data.
Reporting on race, ethnicity, or other socially relevant groupings	We did not collect or report on race or ethnicity.
Population characteristics	Population characteristics are available in Table 1.
Recruitment	Participants were recruited through the three participating sites in Australia (Westmead Hospital and Melanoma Institute Australia in NSW and the Peter MacCallum Cancer Centre in VIC).
Ethics oversight	The study was performed in accordance with the Declaration of Helsinki and Good Clinical Practice guidelines. Patients provided written informed consent. The study protocol was approved by the human research ethics committee at each participating institution.

Note that full information on the approval of the study protocol must also be provided in the manuscript.

Field-specific reporting

Please select the one below that is the best fit for your research. If you are not sure, read the appropriate sections before making your selection.

☒ Life sciences ☐ Behavioural & social sciences ☐ Ecological, evolutionary & environmental sciences

For a reference copy of the document with all sections, see nature.com/documents/nr-reporting-summary-flat.pdf

Life sciences study design

All studies must disclose on these points even when the disclosure is negative.

Sample size	The sample size of 20 patients per arm (N=60) was calculated to determine whether the pathological response rate was ≤5% or ≥20%. If the number of pathological responses was three or more, the hypothesis that pathological response rate ≤5% was rejected with a target error rate of 0.080 and an actual error rate of 0.075. If the number of pathological responses was two or less, the hypothesis that pathological response rate ≥20% was rejected with a target error rate of 0.210 and an actual error rate of 0.206.
Data exclusions	All patients who enrolled in the study (N=60) were included in the analysis.
Replication	No experiments were performed requiring replication.
Randomization	Patients were randomised in a 1:1:1 ratio via a web-based system in permuted blocks (block sizes 6 and 9) and stratified by BRAF V600E versus non-BRAF V600E (i.e., V600K, V600R, V600D, V600M) mutation.
Blinding	The study was open-label.

Reporting for specific materials, systems and methods

We require information from authors about some types of materials, experimental systems and methods used in many studies. Here, indicate whether each material, system or method listed is relevant to your study. If you are not sure if a list item applies to your research, read the appropriate section before selecting a response.

Materials & experimental systems

n/a	Involved in the study	
☒	☐	Antibodies
☒	☐	Eukaryotic cell lines
☒	☐	Palaeontology and archaeology
☒	☐	Animals and other organisms
☐	☒	Clinical data
☒	☐	Dual use research of concern
☒	☐	Plants

Methods

n/a	Involved in the study	
☒	☐	ChIP-seq
☒	☐	Flow cytometry
☒	☐	MRI-based neuroimaging

Clinical data

Policy information about clinical studies

All manuscripts should comply with the ICMJE guidelines for publication of clinical research and a completed CONSORT checklist must be included with all submissions.

Clinical trial registration	ClinicalTrials.gov: NCT02858921
Study protocol	Key sections of the protocol are available in the Supplementary Information file.
Data collection	Data were collected by the principal investigators of the trials.

Outcomes

Primary outcomes:
- Pathological response rate (complete pathological response [pCR] + near-pCR + partial pathological response) (Week 6)
- pCR rate (Week 6)

Secondary outcomes:
- Objective clinical (RECIST v1.1) response rate (Week 6)
- Recurrence-free survival (up to 5 years)
- Event-free survival (up to 5 years)
- Treatment-free survival (up to 5 years)
- Overall survival (up to 5 years)
- Surgical outcomes:
  - Incidence of post-operative infection (Week 6)
  - Incidence of post-operative seroma (Week 6)
  - Duration of post-operative wound drainage time (Week 6)
  - Incidence of post-operative bleeding requiring return to theatre or transfusion (Week 6)
  - Resectability assessment (baseline to Week 6)
- Treatment-emergent adverse events (to Week 52)
- Tissue and liquid biopsy analysis:
  - Characterisation of the immunophenotype of tumour infiltrating cells in melanoma tissue (baseline, Week 1, Week 2, Week 6)
  - Description of the morphological assessment of melanoma tissue (baseline, Week 1, Week 2, Week 6)
  - Description of the RNA expression profile of melanoma tumour (baseline, Week 1, Week 2, Week 6)
  - Measurement of leucocyte subpopulations in peripheral blood (baseline, Week 1, Week 2, Week 6)
  - Measurement of circulating tumour DNA (baseline, Week 1, Week 2, Week 6)

Exploratory outcomes:
- Concordance of metabolic response (FDG PET) measured by pathological response
- Concordance of metabolic response measured by RECIST v1.1 response
- Concordance of pathological response measured by RECIST v1.1 response
- Concordance of metabolic response (FDG PET) with RECIST v1.1 response at relapse
- Concordance of immune-related response criteria (irRC) with RECIST v1.1 response
- Correlation of the gut microbiome with RECIST v1.1 response to immunotherapy
- Characterisation of the bacterial diversity and composition in stool samples
- Characterisation of self-reported dietary habits (including use of oral probiotics) and correlation with the gut microbiome
